# Sequential water molecule binding enthalpies for aqueous nanodrops containing a mono-, di- or trivalent ion and between 20 and 500 water molecules†
†Electronic supplementary information (ESI) available: Detailed description of the experimental and computational modeling methods. Isolation, BIRD and UVPD sequence for [Ru(NH_3_)_6_]^3+^·(H_2_O)_169–171_, nanoESI spectra for 2+ and 3+ ions. Detailed description of the isotope distribution simulation program. Comparison between experimental and simulated 1+, 2+ and 3+ ion isotope distributions. Wavelength dependence of the deduced sequential binding enthalpies. Comparison of experimental UVPD binding enthalpies to the liquid drop model at different temperatures. Complete list of binding enthalpies and average number of water molecules lost upon UVPD. See DOI: 10.1039/c6sc04957e



**DOI:** 10.1039/c6sc04957e

**Published:** 2017-01-26

**Authors:** Sven Heiles, Richard J. Cooper, Matthew J. DiTucci, Evan R. Williams

**Affiliations:** a Department of Chemistry , University of California , Berkeley B42 Hildebrand Hall , Berkeley , California 94720-1460 , USA . Email: erw@berkeley.edu ; Tel: +1-510-643-7161

## Abstract

Sequential water molecule binding enthalpies, Δ*H*_*n*,*n*–1_, are important for a detailed understanding of competitive interactions between ions, water and solute molecules, and how these interactions affect physical properties of ion-containing nanodrops that are important in aerosol chemistry. Water molecule binding enthalpies have been measured for small clusters of many different ions, but these values for ion-containing nanodrops containing more than 20 water molecules are scarce. Here, Δ*H*_*n*,*n*–1_ values are deduced from high-precision ultraviolet photodissociation (UVPD) measurements as a function of ion identity, charge state and cluster size between 20–500 water molecules and for ions with +1, +2 and +3 charges. The Δ*H*_*n*,*n*–1_ values are obtained from the number of water molecules lost upon photoexcitation at a known wavelength, and modeling of the release of energy into the translational, rotational and vibrational motions of the products. The Δ*H*_*n*,*n*–1_ values range from 36.82 to 50.21 kJ mol^–1^. For clusters containing more than ∼250 water molecules, the binding enthalpies are between the bulk heat of vaporization (44.8 kJ mol^–1^) and the sublimation enthalpy of bulk ice (51.0 kJ mol^–1^). These values depend on ion charge state for clusters with fewer than 150 water molecules, but there is a negligible dependence at larger size. There is a minimum in the Δ*H*_*n*,*n*–1_ values that depends on the cluster size and ion charge state, which can be attributed to the competing effects of ion solvation and surface energy. The experimental Δ*H*_*n*,*n*–1_ values can be fit to the Thomson liquid drop model (TLDM) using bulk ice parameters. By optimizing the surface tension and temperature change of the logarithmic partial pressure for the TLDM, the experimental sequential water molecule binding enthalpies can be fit with an accuracy of ±3.3 kJ mol^–1^ over the entire range of cluster sizes.

## Abstract 2

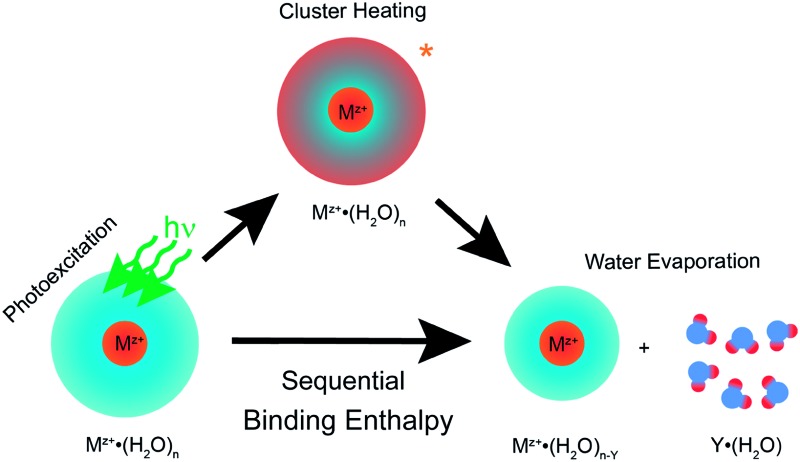
Sequential water binding enthalpies for aqueous nanodrops containing 20–500 water molecules and a 1+ to 3+ ion are reported.

## Introduction

The interactions between water molecules and solute ions can significantly affect the structure, dynamics and reactivity of the solute and the dynamics and the structures of the hydrogen-bonding network of liquid water. Consequently, molecular level knowledge of these interactions is important to understanding processes in solution and in the atmosphere, such as protein folding, molecular recognition and ion-assisted aerosol formation.^1–8^ For example, the protein interlokin-1β contains buried water molecules in the interior that are hydrogen bonded and bridge distant charged amino acid residues by the formation of hydrogen bonds.^5^ It is believed that these ion–water interactions are important for the folding dynamics and structure of the protein. Ions that are formed in the atmosphere are thought to be responsible for the fast nucleation of water molecules in the early stages of aerosol formation, where charged clusters grow faster and become thermodynamically stable at smaller cluster sizes than their neutral counterparts.^7^

One way to obtain detailed information about ion–solvent interactions is to study hydrated ions in a well-defined environment, *i.e.*, clusters of water molecules containing a single ion.^9–27^ In this way, any effects of impurities or counter ions are eliminated. By investigating size selected clusters, it is possible to monitor how thermodynamic quantities, such as water molecule binding enthalpies, evolve with cluster size.^13,28–45^ Several methods have been used to measure the sequential water molecule binding energies of hydrated ions with up to 14 water molecules attached.^28–45^ The most commonly used approaches are high-pressure ion source mass spectrometry (HPMS),^28–34^ threshold collision induced dissociation (TCID)^35–42^ and blackbody infrared radiative dissociation (BIRD).^43–45^ Singly and doubly charged hydrated ions have been studied with these methods. The results reveal that especially for the first hydration shell, the ion identity and charge state significantly influence water molecule binding enthalpies. As the result of increasing ion solvation, the influence of the sequential water binding enthalpies on the specific ion–water interactions diminishes with increasing cluster size. For example, the sequential water molecule binding enthalpies for hydrated Li^+^ decrease from 134 to 63 kJ mol^–1^ for one to six water molecules attached.^37^

Ultraviolet photodissociation (UVPD) experiments have been used to deduce water molecule binding enthalpies at larger cluster sizes and these measurements can be made with high precision. The sequential water binding enthalpies for hydrated aniline, protonated proflavine, protonated tryptophan, rhodamine 590 and rhodamine 640 ions have been studied by UVPD.^46,47^ In these experiments, the sequential water molecule binding enthalpy is deduced from the number of water molecules that are lost from the cluster upon photoexcitation with a photon of known energy. For aniline^+^·(H_2_O)_*n*_ with *n* = 5–20 (where *n* is the number of water molecules) for example, the energy removed per water molecule decreases from 74.5 kJ mol^–1^ at *n* = 5 to an average value of ∼40.2 kJ mol^–1^ between *n* = 10–20.^46^ A very similar trend but slightly higher sequential water molecule binding enthalpies were measured for hydrated doubly charged atomic ions and paraquat.^47,48^ These UVPD studies indicate that for larger cluster sizes, the sequential water molecule binding enthalpies are close to the bulk water vaporization enthalpy (43.1 kJ mol^–1^ (ref. 49)) and depend only slightly on the cluster size. These results and recent velocity map imaging experiments^50^ are consistent with the idea that the absorbed photon energy is fully redistributed into the internal modes of the entire cluster for *n* > 10, resulting in sequential water molecule evaporation, although ion fluorescence resulting in fewer water molecules that are lost can also occur.^47,51^

Despite the progress in obtaining thermodynamic reference data for larger hydrated ions, only limited data for sequential water binding enthalpies over a broad range of cluster sizes and for different charge states is available. These data are especially important to accurately model ion-induced water nucleation^6,7^ in the atmosphere and for ion nanocalorimetry.^9–13^ In the latter method, hydrated ions are irradiated, for example, with slow electrons that can lead to a one-electron reduction of the ion.^9^ The recombination energy (RE), which corresponds to the energy released due to the ion-electron recombination, leads to the evaporation of water molecules from the hydrated ion. The RE can be obtained from the number of water molecules that are lost by modeling of this thermochemical process for water evaporation, which is based on the sequential water molecule binding energies as well as energy that partitions into translational, rotational and vibrational modes of the departing water molecules. By extrapolating REs measured as a function of cluster size to “infinite dilution”, absolute reduction potentials of metal ions can be measured and ultimately related to an absolute reduction potential of the standard hydrogen electrode (SHE).^10,11,13^ Due to the lack of thermochemical reference data for sequential water binding enthalpies of large ion-containing water clusters, the RE modeling currently uses values from the Thomson liquid drop model (TLDM).^52–58^ The TLDM combines the self-energy of solvating a charged particle in the Born solvation model with the increased energy due to the droplet surface area and the bulk vaporization energy to give sequential binding enthalpies of water molecules as a function of cluster size. Any systematic error in the TLDM will result in an error for the absolute SHE value obtained from the ion nanocalorimetry measurements, and this error increases with increasing number of water molecules that are lost as a result of ion-electron recombination.

In this work, we present an extensive study of sequential water binding enthalpies as a function of charge state (*z* = +1–3), ion identity, and cluster size (*n* = 20–500) derived from high-precision UVPD measurements. A newly developed program is used to deduce Δ*H*_*n*,*n*–1_ from experimental precursor and product cluster distributions. The various ions studied for every charge state, the different types of electronic transitions ranging from atomic transitions to electronic excitations in aromatic systems to charge transfer transitions, and the diverse chemical nature of the formed atomic, organic and inorganic ions, make the herein presented binding enthalpy trends important for many chemically relevant systems. The sequential water molecule binding enthalpies show a clear dependence on the ionic charge and cluster size and depend less on ion identity. The results are compared to binding enthalpy predictions from the TLDM. A better agreement between theory and experiment is obtained for the TLDM model employing water ice parameters at 133 K, indicating that the clusters may largely solidify under these experimental conditions.

## Results and discussion

### Ultraviolet photodissociation of hydrated ions

**Fig. 1:**
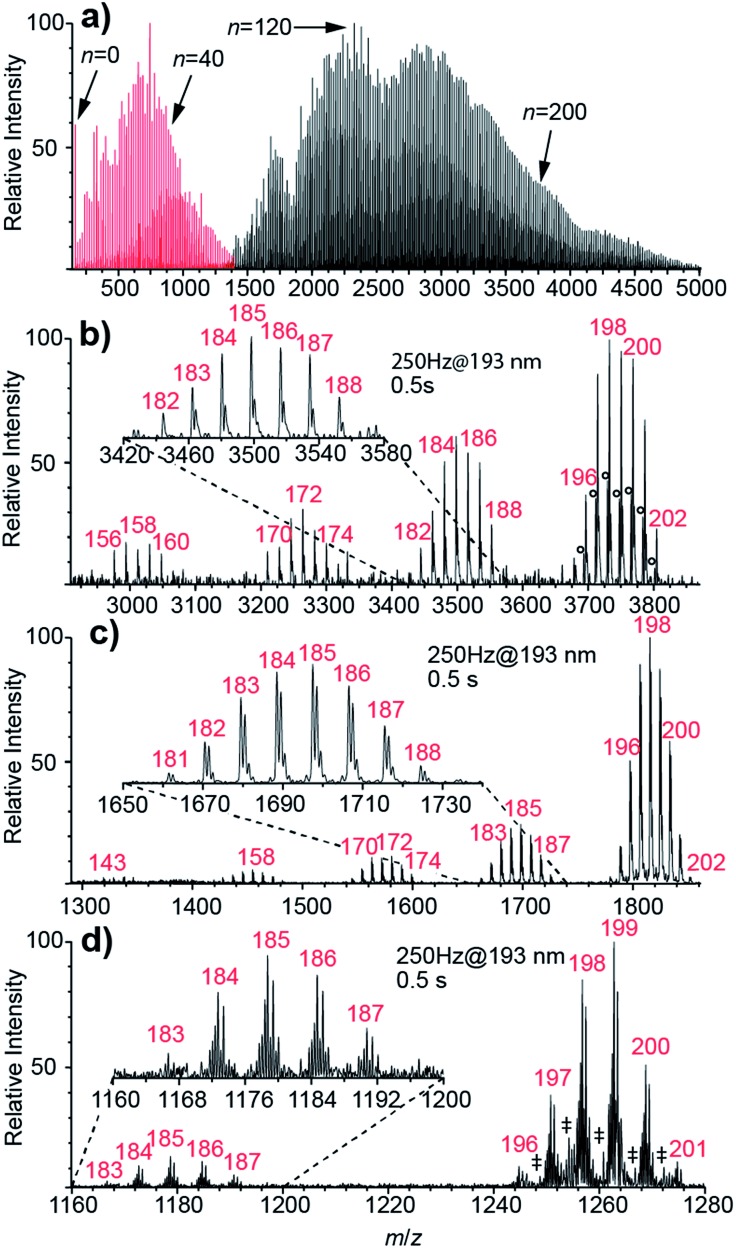
(a) Overlay of two mass spectra of (Phe + H)^+^·(H_2_O)_*n*_ optimized for small (red) and large (black) clusters. Some cluster sizes *n* are highlighted. (b)–(d) UVPD mass spectra of (b) (Phe + H)^+^·(H_2_O)_198–202_, (c) Cu^2+^·(H_2_O)_198–202_ and (d) [Ru(NH_3_)_6_]^3+^·(H_2_O)_198–202_. After 0.5 s of UV radiation with 193 nm laser light at 250 Hz, a loss of ∼13.5 water molecules compared to the precursor cluster size is observed. The delay time of 1.0 s before detection was used in order to eliminate effects of any kinetic shift. The number of water molecules for the selected clusters *n* is shown in red. (○) Unidentified chemical noise; (‡) [Ru(NH_3_)]_6_^2+^·(H_2_O)_*n*_.

**Scheme 1:**
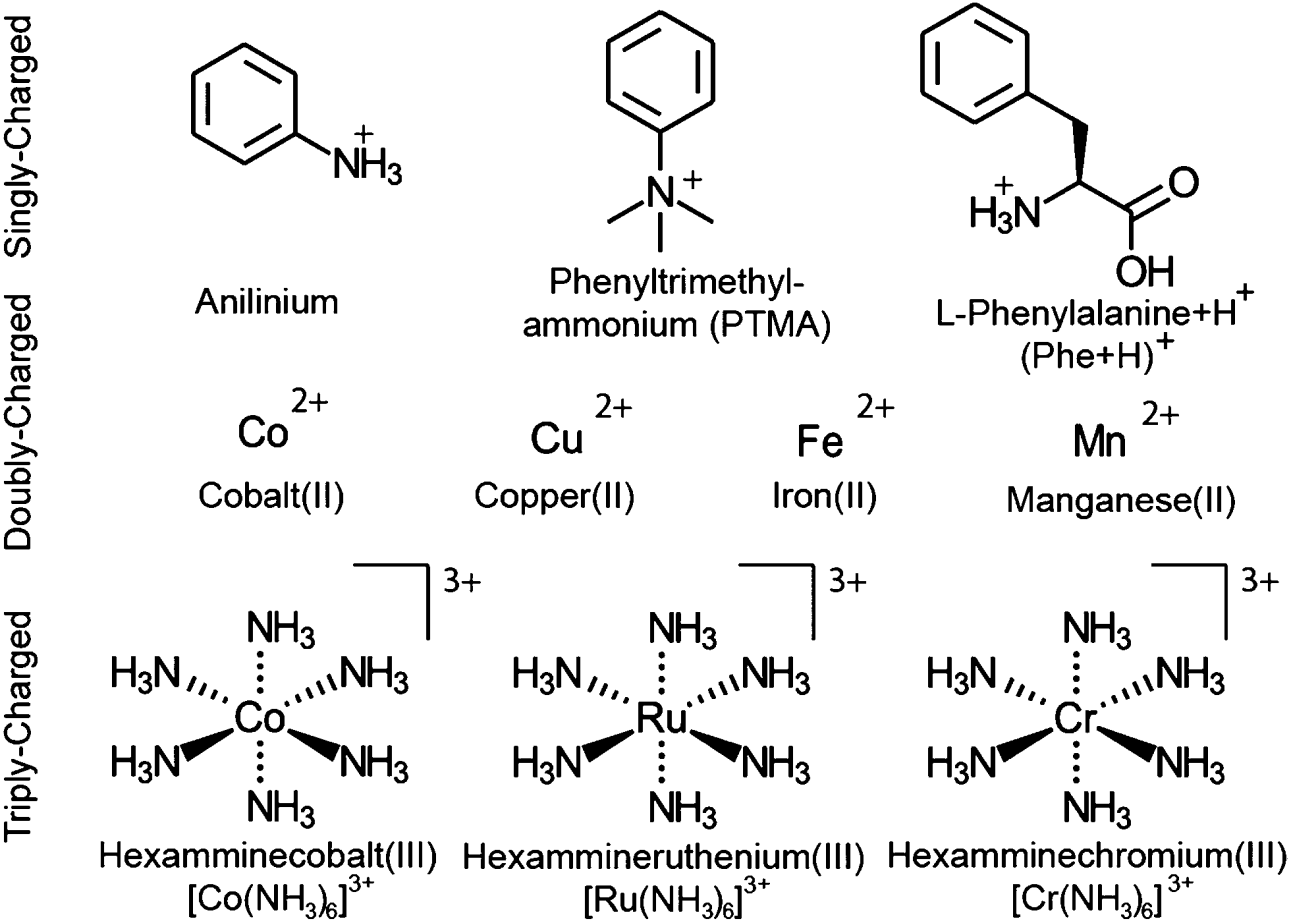
Structures and abbreviations for all ions that are investigated.

Nanoelectrospray ionization coupled to FT-ICR mass spectrometry is used to form and subsequently trap broad distributions of hydrated ions. Two example mass spectra of (Phe + H)^+^ measured using conditions optimized for either small or large cluster sizes are shown in Fig. 1a. Lower heated metal capillary temperatures and lower ion source potentials lead to the formation of larger clusters. Between 0 and ∼250 water molecules are attached to one (Phe + H)^+^ ion, as is the case for other singly charged ions (Scheme 1). For 2+ and 3+ ions, nanoESI mass spectra show hydrated di- and trivalent ions with up to ∼500 and ∼600 water molecules, respectively (see Fig. S2†). Lower detection efficiency at high *m*/*z* limits the cluster sizes that we are able to observe. For divalent ions, the maximum cluster size is about four times greater than that reported previously in UVPD experiments (*n* ≈ 125)^48^ as a result of a higher field strength magnet used in these experiments.

Results of UVPD of (Phe + H)^+^, Cu^2+^ and [Ru(NH_3_)_6_]^3+^ with 193.0 ± 0.5 nm laser light, 250 Hz repetition rate and 0.5 s irradiation time for isolated ensembles with *n* = 198–202 water molecules are shown in Fig. 1b–d, respectively. In addition to the isolated precursors at *n* = 198–202, new clusters with *n* = 195–197, which are formed by BIRD, appear. The mean cluster size, 〈*n*〉, for the precursor distributions shown in Fig. 1b–d containing (Phe + H)^+^, Cu^2+^ and [Ru(NH_3_)_6_]^3+^ ions is 198.73, 198.09 and 198.60, respectively. There are also product ion distributions at lower mass that are generated due to UVPD (Fig. 1b–d). The average cluster size of the highest mass product ion distribution that is separated from the precursor is 185.24, 184.66 and 185.17 for (Phe + H)^+^, Cu^2+^ and [Ru(NH_3_)_6_]^3+^. This product ion distribution has 〈*x*〉 = 13.50, 13.43 and 13.43 fewer water molecules than the corresponding precursor ion. Both the precursor and fragment distributions include contributions from BIRD, which should effect both populations similarly for large clusters.^48^ The difference in the average number of water molecules for these populations should reflect just UV absorption consistent with 〈*x*〉 being independent of the laser irradiation time (see ESI†). For (Phe + H)^+^·(H_2_O)_198–202_ and Cu^2+^·(H_2_O)_198–202_, additional product ion distributions are formed at even lower mass (Fig. 1b and c). For the second and third product ion distribution of (Phe + H)^+^ and Cu^2+^ containing water clusters, an average of 26.46 (26.83) and 40.77 (40.31) water molecules are lost from the precursor ion distribution. For the UVPD mass spectra shown in Fig. 1b and c, the consecutive product ion distributions differ by 13.4–13.5 water molecules. Thus, the absorption of one 193 nm UV photon by ions solvated by ∼200 water molecules results in the loss of about 13.4 water molecules and the formation of product ion distributions with similar hydration state width as the precursor distribution, namely 5–7 hydration states for the precursor and 5–8 for product ion distributions. These multiple product ion distributions (Fig. 1b and c) are due to sequential UV photon absorption, *i.e.*, one, two and three 193 nm UV photons are absorbed sequentially to form the first, second and third product ion distribution, respectively, and not to multiphoton processes. A detailed discussion of sequential UV photon absorption on experimental UVPD results is provided in ref. 48.

Whereas 〈*x*〉 for (Phe + H)^+^·(H_2_O)_198–202_, Cu^2+^·(H_2_O)_198–202_ and [Ru(NH_3_)_6_]^3+^·(H_2_O)_198–202_ do not significantly differ (Fig. 1b–d), the photofragment yield is affected by ion identity. The UVPD yields vary from 51% for (Phe + H)^+^·(H_2_O)_198–202_ to 31% and 12% for Cu^2+^·(H_2_O)_198–202_ and [Ru(NH_3_)_6_]^3+^·(H_2_O)_198–202_, respectively. (Phe + H)^+^ in aqueous solution has a strong absorption band between 190–210 nm as a result of the phenyl group.^59^ The UV absorption at 193 nm for [Ru(NH_3_)_6_]^3+^ is due to a ligand-to-metal charge transfer transition.^60^ The molar extinction coefficient for (Phe + H)^+^ in the water at 193 nm is about six times larger than that for [Ru(NH_3_)_6_]^3+^,^60^ consistent with the higher photoproduct yield for (Phe + H)^+^·(H_2_O)_198–202_ compared to [Ru(NH_3_)_6_]^3+^·(H_2_O)_198–202_. No solution phase reference data is available for Cu^2+^ ions (nor other divalent transition metal ions) at 193 nm precluding a comparison to this ion. However, the photoproduct yield for Cu^2+^·(H_2_O)_198–202_ and other divalent transition metal ions used in this study (Scheme 1) are similar to previously reported results on hydrated divalent ions with *n* ≤ 124.^48^

### Relaxation following UV excitation

**Fig. 2:**
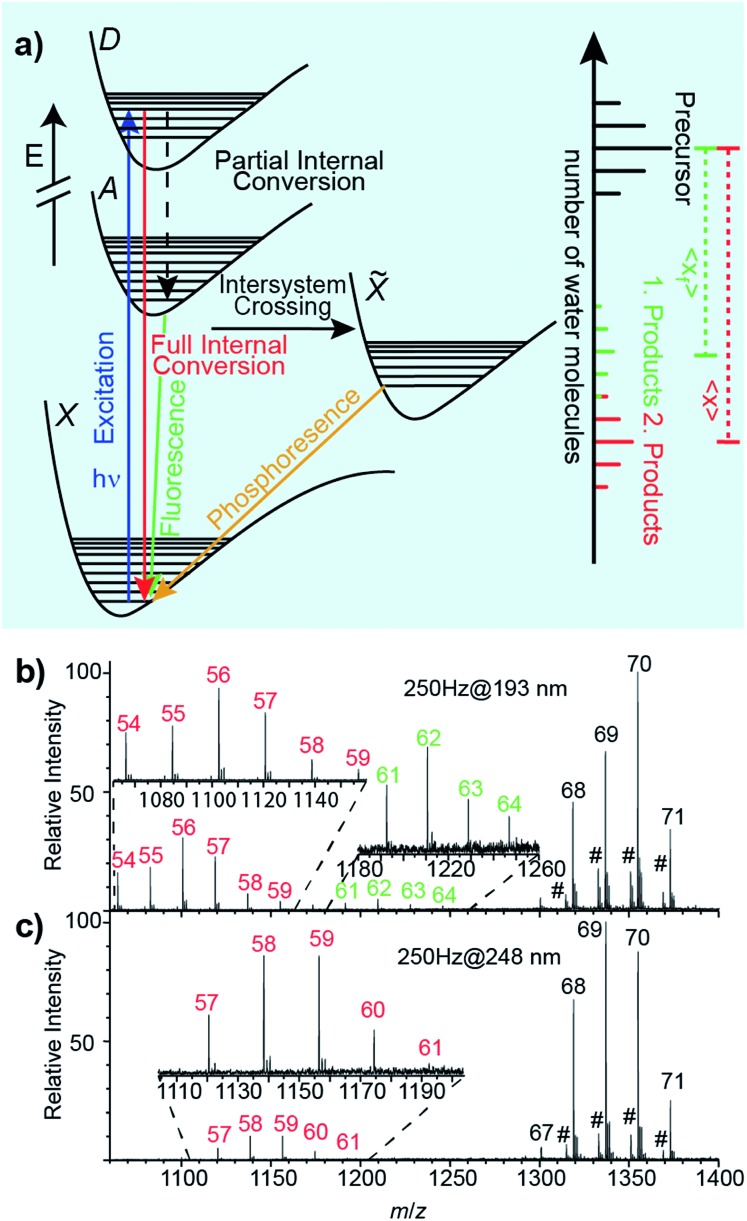
(a) Jablonski diagram of photoexcitation and possible relaxation processes that can occur during UVPD experiments and example precursor and product ion distributions as a result of full internal and partial internal conversion. UVPD mass spectra of anilinium·(H_2_O)_69–71_ at (b) 193 nm and (c) 248 nm. In (b) two product distributions are observed. The distribution centered at 〈*x*〉 ≈ 56 is the result of full internal conversion. The second distribution centered at 〈*n*〉 ≈ 62.5 originates from ions that fluoresce. In (c), there is one UVPD distribution centered at 〈*n*〉 ≈ 58.5 arising from full internal conversion. (#) Unidentified chemical noise.

An energy level diagram of absorption and possible relaxation mechanisms for a water cluster containing a single ion is shown in Fig. 2a. UV photon absorption followed by heat transfer to surrounding water molecules increases the internal energy of the cluster. If radiative emission of a visible or UV photon occurs through fluorescence, less energy is available to convert into internal modes. Thus, the number of water molecules lost when the UV photon that is absorbed and is fully converted into internal modes, 〈*x*〉, is higher than that when partial internal conversion is followed by fluorescence, 〈*x*_f_〉 (Fig. 2a). Additional details of these processes are given in ref. 47 and 48.

(Phe + H)^+^·(H_2_O)_198–202_, Cu^2+^·(H_2_O)_198–202_ and [Ru(NH_3_)_6_]^3+^·(H_2_O)_198–202_ lose ∼13.5 water molecules upon UVPD at 193 nm. Previously reported sequential water molecule binding energies, *E*_*n*,*n*–1_, are ∼0.45 eV per water molecule for clusters with *n* = 10–124.^46,48^ Assuming that water molecules are lost sequentially as a result of full internal conversion from an excited electronic state to the ground electronic state, the number of water molecules that are lost is estimated to be 14.2 for a 6.41 eV photon. This is ∼0.7 water molecules higher than the experimental value of ∼13.5. Because this simple calculation does not take into account energy partitioned into the degrees of freedom of the water molecules that evaporated, this calculation overestimates the number of water molecules that are lost. Hence, our experimental results are consistent with water loss due to full internal conversion of the absorbed photon. Further evidence for sequential water molecule loss and full internal conversion producing the product distributions at about 〈*x*〉 = *hν*/*E*_*n*,*n*–1_ below the precursor distribution in UVPD experiments of hydrated ions comes from the wavelength-dependent UVPD measurements of anilinium·(H_2_O)_69–71_ shown in Fig. 2b and c. In the 193 nm UVPD experiment shown in Fig. 2b, there are two product ion distributions. The distribution corresponding to the most extensive water loss is shifted by 〈*x*〉 = 13.47 water molecules compared to the precursor ion distribution whereas the other distribution is shifted by 〈*x*_f_〉 = 7.22. The product distribution with the highest water loss is consistent with the expected loss of ∼13.5 water molecules due to full internal conversion of a 193 nm photon, and the additional product ion distribution is due to partial internal conversion followed by fluorescence with an emitted photon wavelength of ∼2.80 eV.^47^ The fluorescence quantum yield, obtained from the relative ion abundances of the two product ion distributions, is 0.11. UVPD of anilinium·(H_2_O)_69–71_ at 248 nm results in only a single product ion distribution corresponding to 〈*x*〉 = 10.68 (Fig. 2c). For the photon energy of 5.0 eV (248 nm), an estimated water loss of 〈*n*〉 = 11.11 is expected from full internal conversion, consistent with the experimental result. These measurements show that the fluorescent quantum yield varies significantly with excitation photon wavelength.

Because the number of water molecules lost from precursor ion distributions to product ion distributions at ∼*hν*/*E*_*n*,*n*–1_ are consistent with a full internal conversion process and additional ion product distributions are identified for partial internal conversion/fluorescence processes, we conclude that full internal conversion and sequential water loss are the major processes leading to product ion distributions at 〈*x*〉 ≈ *hν*/*E*_*n*,*n*–1_ in UVPD.

### Kinetic shift effect

**Fig. 3:**
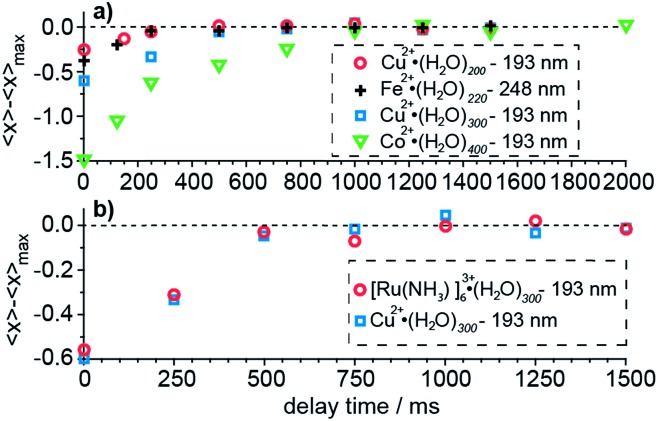
The dependence of the average number of water molecules lost, 〈*x*〉, minus the maximum number of water molecules lost, 〈*x*_max_〉, as a function of the delay time between photoexcitation and ion detection (the kinetic shift). 〈*x*〉 for the largest delay time was set to 〈*x*_max_〉 as indicated by the dashed line. The median of the selected cluster ensemble is used to label the clusters. (a) Kinetic shift as a function of cluster size and wavelength. (b) Kinetic shift as a function of charge state for [Ru(NH_3_)_6_]^3+^·(H_2_O)_300_ and Cu^2+^·(H_2_O)_300_.

The time required for all of the water molecules that will ultimately evaporate from the cluster following absorption of a UV photon depends on the cluster size. The time necessary for evaporation of the water molecules increases with increasing cluster size owing to an increasing number of degrees of freedom over which this energy is distributed.^61^ The potential influence of measurement time on the product ion cluster size is a kinetic shift effect. The extent to which a kinetic shift affects these measurements is measured by varying the delay time between laser irradiation and ion detection until the maximum number of water molecules, 〈*x*_max_〉, that are lost, is observed. Results of these kinetic shift measurements as a function of cluster size, charge state and laser wavelength are shown in Fig. 3. Our results for hydrated divalent ions show an increasing kinetic shift with increasing cluster size for clusters with *n* ≥ 200 (Fig. 3a). At zero detection delay time, 〈*x*〉 for Cu^2+^·(H_2_O)_198–202_, Cu^2+^·(H_2_O)_298–302_ and Co^2+^·(H_2_O)_398–402_ (labeled as the median of the cluster ensemble in Fig. 3) are lower than 〈*x*_max_〉 by 0.23, 0.58 and 1.50 water molecules. For Cu^2+^·(H_2_O)_198–202_, Cu^2+^·(H_2_O)_298–302_ and Co^2+^·(H_2_O)_398–402_, a delay time between photoexcitation and ion detection of 250 ms, 500 ms and 1000 ms are required so that 〈*x*〉 approaches 〈*x*_max_〉 to within 0.05 water molecules. The kinetic shift does not depend significantly on laser wavelength within this range of photon energies (Fig. 3a). At zero detection delay time, Cu^2+^·(H_2_O)_198–202_ at 193 nm and Fe^2+^·(H_2_O)_218–222_ at 248 nm lose 0.23 and 0.39 water molecules less than 〈*x*_max_〉 and both approach 〈*x*_max_〉 at about 250 ms. The independence of the kinetic shift on laser wavelength is consistent with the time necessary for the water evaporation process to occur being mainly limited by the last water molecule that is lost from the cluster, which depends primarily on cluster size (degrees of freedom) and not on the initial energy deposited. The bigger difference at zero detection delay for the iron compared to the copper containing water cluster is consistent with the slightly larger cluster size of the former. The charge state dependence of the kinetic shift is investigated for Cu^2+^·(H_2_O)_298–302_ and [Ru(NH_3_)_6_]^3+^·(H_2_O)_298–302_ at 193 nm (Fig. 3b). All data points for different delay times differ by less than 0.09 water molecules for the two clusters indicating that the charge state does not significantly contribute to the kinetic shift effect. We conclude that the kinetic shift increases with increasing cluster size but not notably with excitation wavelength and charge state. A detection delay time after laser irradiation of 500 ms, 1000 ms and 1500 ms for *n* < 100, 100 ≤ *n* ≤ 300 and *n* > 300 in our UVPD measurements, respectively, eliminates effects of the kinetic shift on the 〈*x*〉 and Δ*H*_*n*,*n*–1_ values obtained from these measurements.

### Effects of cluster size, ion charge and ion identity

**Fig. 4:**
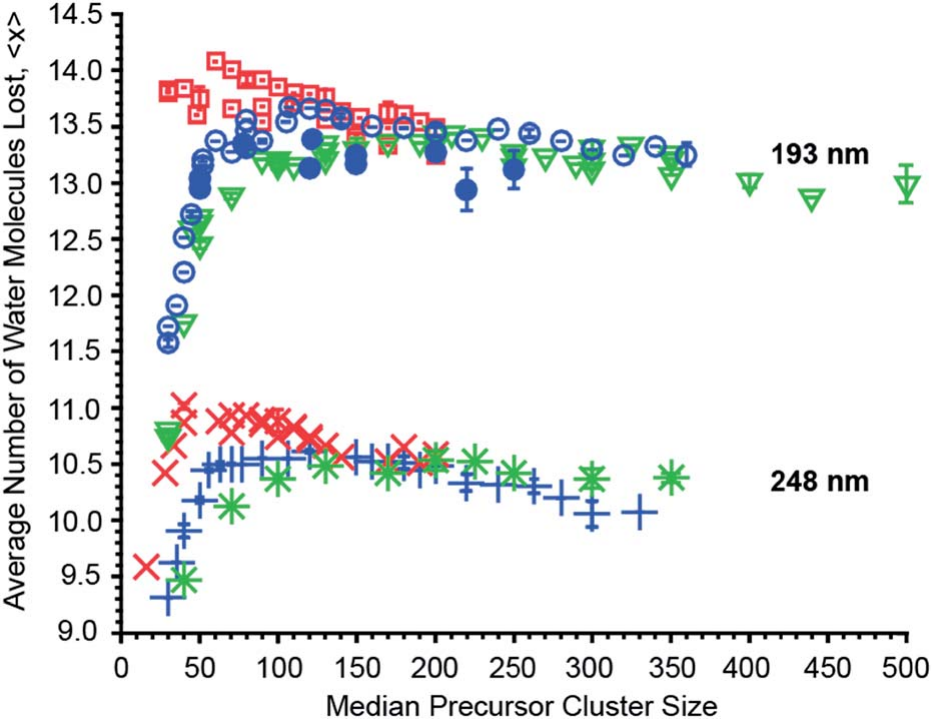
Average number of water molecules lost by hydrated PTMA, (Phe + H)^+^, anilinium (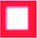); Cu^2+^, Co^2+^ (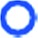); Fe^2+^, Mn^2+^ (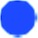); [Co(NH_3_)_6_]^3+^, [Cr(NH_3_)_6_]^3+^, [Ru(NH_3_)_6_]^3+^ (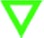) ions upon 193 nm photon absorption and (Phe + H)^+^, anilinium (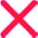); Fe^2+^ (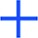); [Ru(NH_3_)_6_]^3+^ (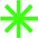) upon 248 nm photon absorption as a function of median precursor cluster size. Error bars indicate the standard deviation of triplicate measurements.

The effects of cluster size, ion charge and ion identity on the number of water molecules lost from different hydrated ions upon 193 nm and 248 nm photon absorption were measured as a function of cluster size and these data are shown in Fig. 4. The number of water molecules lost following photoabsorption depends on each of these factors to differing extents. For example, the number of water molecules lost upon 193 nm UVPD increases from a minimal value of 10.74 ± 0.03 for [Ru(NH_3_)_6_]^3+^ with a median of 30 water molecules attached to a maximum of 14.01, 13.67 and 13.44 for mono-, di- and trivalent ions, which occurs at median cluster sizes of 70, 110 and 210, respectively. Beyond the maxima, the number of water molecules that are lost decreases monotonically with cluster size. Similar changes in 〈*x*〉 with cluster size also occur for 248 nm UVPD. With 248 nm photons, 〈*x*〉 is about three water molecules less than that with 193 nm photons, but the qualitative dependence of 〈*x*〉 with median cluster size follows the same trends as for the 193 nm data (Fig. 4).

In addition to cluster size, the charge state of the hydrated ion affects the average number of water molecules that are lost (Fig. 4). For clusters of the same median size, 〈*x*〉 for monovalent ions is bigger than that for divalent ions which is bigger than that for trivalent ions up to a cluster size ∼150. This is consistent with the TLDM model that predicts this charge-dependent ordering of *E*_*n*,*n*–1_.^52^ The differences in the number of water molecules that are lost deceases significantly for clusters with more than ∼150 water molecules (Fig. 4). This indicates a vanishing influence of the charge state on the binding energies of water molecules to the large clusters.

The identity of the ion of a given charge state and at the same cluster size has only a small effect on the number of water molecules lost (Fig. 4). For example, [Ru(NH_3_)_6_]^3+^ and [Co(NH_3_)_6_]^3+^ with 100 water molecules or [Ru(NH_3_)_6_]^3+^ and [Cr(NH_3_)_6_]^3+^ with 350 water molecules lose 13.21 ± 0.03 and 13.16 ± 0.03 or 13.21 and 13.26 water molecules at 193 nm excitation wavelength. Similarly, 〈*x*〉 differs only by 0.02 for 248 nm UVPD of (Phe + H)^+^ and anilinium with 120 water molecules attached. Although the number of water molecules lost following photoabsorption is minimally affected by ion identity for the majority of the ions investigated, 〈*x*〉 at 193 nm for hydrated Mn^2+^ and Fe^2+^ ions differs significantly from that for Cu^2+^ and Co^2+^ at precursor sizes with *n* > 130. At a precursor ensemble size of 220, 〈*x*〉 is 13.38 ± 0.04 and 12.94 ± 0.19 for Cu^2+^ and Fe^2+^, respectively. More water molecules can be lost from a cluster either as a result of lower water molecule binding energies or competition between water molecule loss and internal energy conversion. If internal conversion is not instantaneous, ions are not heated to as high of an effective temperature compared to when internal conversion is instantaneous because water molecules that evaporate during internal conversion take away energy. Thus, less energy partitions into translational, rotational and vibration energy of evaporated water molecules. There is evidence for a long-lived excited state for Cu^2+^ in smaller nanodrops for which competition between water loss and internal conversion could be relevant, but this effect should be negligible for the larger clusters investigated here owing to a slower water molecule evaporation rate.^10,62^ Thus, the different number of water molecules lost for Cu^2+^/Co^2+^ compared to Fe^2+^/Mn^2+^ likely reflect a difference in water molecule binding enthalpy. A comparison between the divalent ions at 248 nm is not possible because UVPD products are only observed for Fe^2+^. However, the decrease of 〈*x*〉 for hydrated Fe^2+^ at 248 nm with increasing cluster size, even below 〈*x*〉 for trivalent ions, is also consistent with an increasing binding enthalpy with increasing cluster size (Fig. 4). This is unexpected because the influence of ion identity should decrease with increasing cluster size.^47,48^ A possible explanation for this effect is an ion specific water patterning effect, so that Fe^2+^/Mn^2+^ containing water clusters form water clusters differing in shape or water phase from Cu^2+^/Co^2+^·(H_2_O)_*n*_. Evidence for the influence of ions on the water phase in large water clusters comes from a recent study that showed that La^3+^ ions can affect the onset of crystallinity in clusters as large as 375 water molecules.^63^

### Effective cluster temperatures and kinetic energy release

**Fig. 5:**
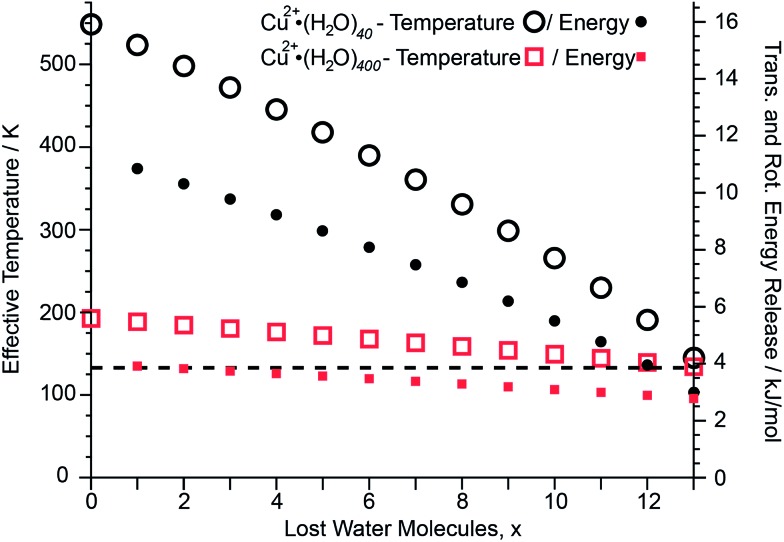
Effective cluster temperature (open symbols) and translational/rotational energy (filled symbols) release for Cu^2+^·(H_2_O)_40_ (black) and Cu^2+^·(H_2_O)_400_ (red) as a function of water molecules that are lost from the cluster, 〈*x*〉, upon absorption of a 193 nm photon. The dashed black line indicates the experimental temperature of 133 K.

After absorption of one UV photon and full internal conversion back to the electronic ground state of an ion, the effective temperature of the cluster increases from its initial effective temperature of ∼133 K established by the interaction of the ions with the blackbody radiation field inside the ion cell prior to photoexcitation. Absorption of a photon shifts the initial internal energy distribution of the cluster by a value corresponding to the energy of the photon. The extent of this effective temperature increase depends on the cluster size and the energy of the absorbed photon and is modeled as described in the ESI.† This effect is illustrated for two different Cu^2+^ cluster sizes (Fig. 5). Upon absorption of a 6.41 eV photon and internal conversion, the initial effective temperature of Cu^2+^·(H_2_O)_40_ and Cu^2+^·(H_2_O)_400_ prior to any water molecule loss increases to ∼550 K and ∼190 K, respectively. Both clusters cool back down to ∼130 K after 13 water molecules are lost. The higher initial effective temperature of Cu^2+^·(H_2_O)_40_ compared to Cu^2+^·(H_2_O)_400_ is due to the lower number of degrees of freedom of the smaller cluster. The energy partitioned into the degrees of freedom for the sequentially evaporated water molecules depends on the effective cluster temperature at which each water molecule is lost (ESI eqn (3)†). Consequently, the lower effective temperature for Cu^2+^·(H_2_O)_400_ than that for Cu^2+^·(H_2_O)_40_ results in less 〈*E*_VRT_〉 (see Fig. 5; 〈*E*_VRT_〉 = 8.16 and 3.68 kJ mol^–1^ for Cu^2+^·(H_2_O)_40_ and Cu^2+^·(H_2_O)_400_, respectively). The sequential water molecule binding energies for Cu^2+^·(H_2_O)_40_ and Cu^2+^·(H_2_O)_400_ obtained by modeling the water molecule loss are 39.37 kJ mol^–1^ and 43.85 kJ mol^–1^, respectively. The relative energy contribution from the total energy release of 8.16 and 3.68 kJ mol^–1^ for Cu^2+^·(H_2_O)_40_ and Cu^2+^·(H_2_O)_400_ to the deposited energy of 6.41 eV is 17% and 8%, respectively, and decreases with increasing cluster size. Thus, any systematic error in the computed binding energies introduced by using this energy release model decreases with cluster size. For mono-, di- and trivalent clusters with up to 350 water molecules, the difference between calculated binding energies obtained from 248 nm and 193 nm UVPD experiments are shown in Fig. S6.† The mean difference between 248 nm and 193 nm binding energies is 0.4 ± 0.6 kJ mol^–1^. The average difference of 0.4 kJ mol^–1^ for 248 nm compared to 193 nm binding energies indicates that the model may slightly overestimate the kinetic energy release, but this difference is well within the uncertainty of our measurements. This indicates that the kinetic energy release model introduces no significant systematic error for the calculation of average sequential binding enthalpies from UVPD experiments.

### Average water molecule binding enthalpies and the liquid drop model

**Fig. 6:**
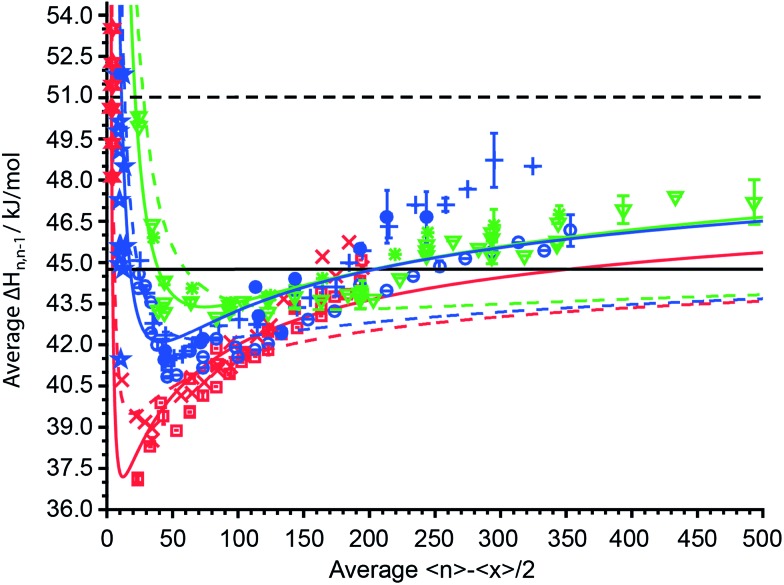
The average sequential water molecule binding enthalpy, Δ*H*_*n*,*n*–1_, in kJ mol^–1^ deduced from UVPD measurements for hydrated PTMA, (Phe + H)^+^, anilinium (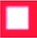); Cu^2+^, Co^2+^ (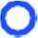); Fe^2+^, Mn^2+^ (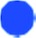); [Co(NH_3_)_6_]^3+^, [Cr(NH_3_)_6_]^3+^, [Ru(NH_3_)_6_]^3+^ (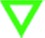) ions upon 193 nm photon absorption and (Phe + H)^+^, anilinium (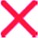); Fe^2+^ (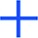); [Ru(NH_3_)_6_]^3+^ (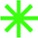) upon 248 nm photon absorption as a function of 〈*n*〉 – 〈*x*〉/2. The sublimation enthalpy Δ*H*_sub_ = 51.0 kJ mol^–1^ of bulk water ice at 133 K and the vaporization enthalpy Δ*H*_vap_ = 44.8 kJ mol^–1^ of bulk water at 273 K are depicted as dashed and solid black horizontal lines, respectively. The TLDM at 133 K and the fitted TLDM are shown as dashed and solid red, blue and green lines for mono-, di-, and trivalent ions, respectively. Literature binding enthalpies for clusters with 〈*n*〉 – 〈*x*〉/2 ≤ 12 monovalent (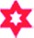) and divalent (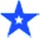) ions are included in the figure.^32–45^

The average sequential binding enthalpies for mono-, di- and trivalent ions obtained from these UVPD experiments, along with previously published data for clusters with fewer than 15 water molecules^32–45^ as a function of 〈*n*〉 – 〈*x*〉/2 are shown in Fig. 6. The uncertainty in size and enthalpy of the Δ*H*_*n*,*n*–1_ values are ±〈*x*〉/2 = ±5.5–7.5 water molecules and ±0.4–1.2 kJ mol^–1^, respectively. The average binding enthalpies for clusters with 20 to 500 attached water molecules decrease for the smallest investigated clusters with increasing cluster size until a minimum in Δ*H*_*n*,*n*–1_ is reached (Fig. 6). Namely, Δ*H*_*n*,*n*–1_ for mono-, di- and trivalent ions decrease from 40.75 kJ mol^–1^, 45.06 kJ mol^–1^ and 50.3 ± 0.16 kJ mol^–1^ for the investigated cluster with the least number of water attached to a minimum of 37.09 ± 0.04 kJ mol^–1^, 40.83 ± 0.03 kJ mol^–1^ and 43.01 kJ mol^–1^ that is located around 〈*n*〉 – 〈*x*〉/2 ≈ 15, 45 and 75, respectively. Beyond the minimum, the binding enthalpies increase with increasing cluster size reaching 44.32 ± 0.20 kJ mol^–1^, 46.17 ± 0.58 kJ mol^–1^ and 47.22 ± 0.81 kJ mol^–1^ for (Phe + H)^+^·(H_2_O)_198–202_, Cu^2+^·(H_2_O)_358–362_ and [Co(NH_3_)_6_]^3+^·(H_2_O)_498–502_, respectively. The average water molecule binding enthalpies of hydrated Cu^2+^/Co^2+^ ions approach a value of ∼46.0 kJ mol^–1^ at large cluster size, whereas that for hydrated Fe^2+^/Mn^2+^ ions is 48.53 kJ mol^–1^ (Fig. 6). For 〈*n*〉 – 〈*x*〉/2 = 40–125, the average binding enthalpy of divalent ions is 42.3 ± 0.8 kJ mol^–1^, consistent with a previously reported value of 43.1 ± 0.4 kJ mol^–1^ ions with this same charge state and within this same cluster size range.^48^ Although Δ*H*_*n*,*n*–1_ depends on the charge state for small clusters, the average water molecule binding enthalpies converge towards the same values within ±0.63 kJ mol^–1^ for clusters with ∼150 or more water molecules. The only exceptions are Δ*H*_*n*,*n*–1_ values for Fe^2+^/Mn^2+^ ions that are up to 2.5 kJ mol^–1^ above the average binding enthalpies of all other ions. This shows that high accuracy UVPD measurements are able to detect specific ion effects on Δ*H*_*n*,*n*–1_ up to cluster sizes of ∼300. The binding enthalpies for all charge states are below the sublimation enthalpy of bulk ice at 133 K (Δ*H*_sub_ = 51.0 kJ mol^–1^) but exceed the enthalpy of vaporization at 273 K (Δ*H*_vap_ = 44.8 kJ mol^–1^) for large cluster size.^49^

Also shown in Fig. 6 (dashed colored lines) are the average binding enthalpies calculated with the TLDM (see ESI†) using 133 K bulk ice parameters (Table 1). The mean deviation of the TLDM at 133 K with ice parameters for mono-, di- and trivalent ions is lower than the mean deviations (root-mean-square-deviation; RMSD) of the TLDM at 273 K, 298 K and 313 K using liquid water parameters (see ESI†). Namely, the RMSD for monovalent ions is 4.69 kJ mol^–1^, 2.47 kJ mol^–1^, 2.01 kJ mol^–1^ and 1.38 kJ mol^–1^ at 313 K, 298 K, 273 K and 133 K, respectively. The use of ice parameters for the TLDM calculations is consistent with recent results, which indicate that large clusters are “ice-like” at low temperature.^63–66^ It is still debated at what cluster size the thermodynamic concept of phase is applicable to nanodrops and it has been shown that spectroscopic features for amorphous and crystalline ice can coexist for clusters with up to 550 water molecules,^63^ hence, we use the phrase “ice-like” in what follows as a synonym for the presence of amorphous or crystalline ice phases.

**Table 1:** Bulk water properties at 133 K^*a*^

Parameters	133 K
*M*/g mol^–1^	18.015
*ρ*/kg m^–1^	931.7^*b*^
∂*ρ*/∂*T*/kg m^–3^ K^–1^	–0.065^*b*^
*ε*	197.4^*c*^
∂*ε*/∂*T*/K^–1^	–1.5300^*c*^
*γ*/mN m^–1^	109^*d*^
∂*γ*/∂*T*/mN m^–1^ K^–1^	–0.1407^*e*^
ln(*p*/*p*_0_)	–28.6471^*b*^
∂ln(*p*/*p*_0_)/∂*T*/K^–1^	0.3185^*b*^

^*a*^ ref. 52.

^*b*^ ref. 49.

^*c*^ ref. 69.

^*d*^ ref. 70.

^*e*^Not known; 273 K parameter used; value only marginally effect the results.

Although the TLDM using 133 K bulk ice parameters provides binding enthalpies that are more similar to the experimental values for small clusters compared to those using liquid water parameters, the model does not accurately account for the increase of Δ*H*_*n*,*n*–1_ with cluster size above 〈*n*〉 – 〈*x*〉/2 ≈ 175. In order to improve the agreement between values from the TLDM and the experiment at large cluster size changing the least number of parameters in the model, a sensitivity analysis was performed. The surface energy, *γ*, and the change of the logarithmic partial pressure with temperature, ∂ln(*p*/*p*_0_)/∂*T* (*p*_0_ is the standard pressure), have the biggest effect on the TLDM at large cluster size. For mono-, di- and trivalent ions, these two parameters were optimized and the resulting fits are shown in Fig. 6 (solid colored lines). The corresponding charge-dependent parameters are shown in Table 2. The RMSD values improve from 1.25 kJ mol^–1^, 1.92 kJ mol^–1^ and 2.05 kJ mol^–1^ for the 133 K TLDM to 0.92 kJ mol^–1^, 1.17 kJ mol^–1^ and 0.67 kJ mol^–1^ for the corresponding fits for mono-, di- and trivalent ions, respectively. The surface energy and ∂ln(*p*/*p*_0_)/∂*T* increase for all charge states compared to bulk 133 K parameters and the extent of the increase depends on charge. For example, the surface tension for divalent ions is 215 mN m^–1^, which is nearly double the corresponding bulk value. Excluding the Fe^2+^/Mn^2+^ data for the fit of the TLDM for divalent ions, *i.e.*, only using Δ*H*_*n*,*n*–1_ values for Cu^2+^ and Co^2+^ (Table 2 values in parentheses), results in a value of 180 mN m^–1^ for *γ* (Fig. S10†). The higher value of *γ* compared to bulk parameters of pure water ice and the increase in this value with charge state is consistent with the influence of ions on surface energy in electrolyte solutions.^67^ In bulk solutions, the relative surface energy increase of dilute electrolyte solutions compared to pure water is between 0–15% for salts such as K_2_SO_4_ and up to 100–160% for LaCl_3_ or K_4_[Fe(CN)_6_].^67^ Even though these clusters contain only one isolated cation and up to 500 water molecules, solution data provide support for the use of a higher surface energy in the TLDM that should be charge dependent. This indicates that hydrated ions can influence the properties of water, such as surface energy, in clusters containing up to 500 water molecules. The higher ∂ln(*p*/*p*_0_)/∂*T* for clusters compared to bulk water is in qualitative agreement with the Kelvin equation, which predicts an increase of droplet partial pressure compared to bulk water.^68^

**Table 2:** Optimized surface energy, *γ*, and ∂ln(*p*/*p*_0_)/∂*T* in the TLDM for 133 K using Δ*H*_*n*,*n*–1_ values for clusters with 20–500 water molecules and compared to bulk parameters. The RMSD between of experiment and theory is given in kJ mol^–1^. For divalent ions the results in parentheses indicate the fit without Fe^2+^/Mn^2+^ ions

Charge	*γ*/mN m^–1^	∂ln(*p*/*p*_0_)/∂*T*/10^–2^ K^–1^	RMSD/kJ mol^–1^
1+	182 ± 23	34.3 ± 0.7	0.92
2+	215 ± 26 (180 ± 27)	35.5 ± 0.8 (34.1 ± 0.8)	1.17 (0.84)
3+	269 ± 17	36.4 ± 0.5	0.67
Bulk	109^*a*^	31.8^*b*^	—

^*a*^ ref. 69.

^*b*^ ref. 49.

Although optimization of the 133 K TLDM results in an improved fit to the experimental data, it is difficult to interpret the physical relevance of the extracted parameters. The values of *γ* and ∂ln(*p*/*p*_0_)/∂*T* are averaged over all cluster sizes between *n* = 20–500 so there is no explicit size dependence of these parameters. The TLDM also has difficulties in accurately reproducing Δ*H*_*n*,*n*–1_ for small hydrated ions because specific bonding in the first and second solvation shells is not taken into account by the TLDM.^52^ Finally, large and hydrophobic ions like (Phe + H)^+^ or PTMA may not be fully solvated or the nanodrop may not be spherical at small cluster sizes. Despite these factors, the close agreement with experimental data justifies the use of the modified TLDM. Additionally, the relatively close correspondence of the optimized parameters to the bulk ice parameters at 133 K, considering the discussed uncertainties, is consistent with isolated ions in larger nanodrops as “ice-like” particles in the gas phase.

## Conclusions

High-resolution UVPD results as a function of charge state and cluster size for a diverse set of ion-containing nanodrops containing up to 500 water molecules, which include data for trivalent ions for the first time, are presented. Average sequential water molecule binding energies are obtained with ±0.8 kJ mol^–1^ precision. These data have a minimum in Δ*H*_*n*,*n*–1_ at around 75, 45 and ∼15 water molecules for 3+, 2+ and 1+, respectively. This minimum in the binding enthalpies is predicted by the TLDM and stems from the counteracting energetic contributions of the surface and solvation energies. Therefore, these results are consistent with all qualitative predictions of the TLDM. However, the precision of the UVPD results reveal that the unmodified TLDM does not adequately fit the experimental data and the closest agreement between experimental and TLDM values for Δ*H*_*n*,*n*–1_ is achieved using 133 K parameters. Indications for a water–ice phase transition for water clusters with 60–79 water molecules at 133 ± 6 K and for a phase transition to crystalline ice for La^3+^·(H_2_O)_375_ at 133 K have been reported previously.^63–66^ Therefore, our interpretation of the increased average water molecule binding enthalpies within a modified 133 K TLDM is consistent with these findings. With bulk ice parameters and optimizing *γ* and ∂ln(*p*/*p*_0_)/∂*T*, the experimental values can be reproduced with accuracies of ±0.8 kJ mol^–1^ between *n* = 20–500 water molecules. Even though the TLDM is a very simplified model of these complex systems, it does a remarkable job in qualitatively explaining the experimental results. The physical significance of the optimized *γ* and ∂ln(*p*/*p*_0_)/∂*T* values is difficult to evaluate, but the agreement between experimental Δ*H*_*n*,*n*–1_ values and the TLDM with modified ice parameters indicates that the larger clusters are “ice-like” under our experimental conditions. These binding enthalpies can serve as valuable reference values for simulations of ion-induced nucleation. Additionally, values will further improve the accuracy and precision of absolute reduction potential values deduced from ion nanocalorimetry measurements of ions in nanodrops.
