# Supplementary material for: Sequential water molecule binding enthalpies for aqueous nanodrops containing a mono-, di- or trivalent ion and between 20 and 500 water molecules
Source: Chem Sci. 2017 Jan 26;8(4):2973–82. doi: 10.1039/c6sc04957e (PMC5380113; doi:10.1039/c6sc04957e)
Supplement: Supplementary file 1 [file SC-008-C6SC04957E-s001.pdf]

Electronic Supplementary Material (ESI) for Chemical Science.
This journal is © The Royal Society of Chemistry 2017

Sequential Water Molecule Binding Enthalpies for Aqueous Nanodrops Containing a Mono-, Di- or Trivalent Ion and Between 20 and 500 Water Molecules

Sven Heiles, Richard J. Cooper, Matthew J. DiTucci and Evan R. Williams

Department of Chemistry, University of California, Berkeley, California 94720-1460

Supporting Information

Methods

Mass Spectrometry and UVPD Experiments. All experiments were performed on a home-built 7 T Fourier transform ion cyclotron resonance (FT-ICR) mass spectrometer, which is based on the 2.75 T instrument described previously.[1] A schematic diagram of the experimental setup for UVPD experiments is shown in **Figure S12**. Briefly, ions are generated with a home-built nanoelectrospray ionization (nanoESI) interface (see **Figure S12**).[2] Freshly prepared 3-5 mM solutions of phenyltrimethylammonium (PTMA), $CuSO_4$, $CoSO_4$, $FeCl_2$, $MnCl_2{\cdot}(H_2O)_4$ $[Co(NH_3)_6]Cl_3$, $[Ru(NH_3)_6]Cl_3$ and $[Cr(NH_3)_6](NO_3)_3$ in purified water (Milli-Q-purification, Millipore, MA, U.S.A.) are loaded into borosilicate capillaries pulled to an inner tip diameter of ~1 µm. Electrical contact with the sample solution is made with a platinum wire. For aniline and phenylalanine (Phe), 3 – 5 mM solutions in 90/10 water/methanol are acidified with 1% acetic acid. All samples are from Sigma-Aldrich (St. Louis, MO) except for $[Cr(NH_3)_6](NO_3)_3$, which was synthesized as described in the literature.[3] A voltage of ~700 V with respect to the heated entrance capillary is applied to the platinum wire, producing a spray of ion-containing aqueous nanodrops that enter the apparatus (see **Figure S12**; nanoESI Source). A combination of turbomolecular pumps (TP) and helium cryogen pumps (CP) are used (**Figure S12**) to maintain a high vacuum. The hydrated ions are gently guided by electrostatic lenses through five stages of differential pumping into the FT-ICR cell, which is in the center of a 7 T superconducting magnet. The cylindrical Penning trap, which has been described previously,[4] is surrounded by a copper jacket thermalized to 133 K by a regulated flow of liquid nitrogen for at least 8 h prior to experiments (see **Figure S12**; N_2 lines). Ions entering the cell collide with dry nitrogen

gas, which is injected into the cell chamber at a pressure of ~10^{-6} Torr prior to the introduction of the ions into the cell (see **Figure S12**; Gas Inlet). Ion–N_2 collisions assist the trapping and thermalization of the ions. After 4 – 10 s of ion accumulation, a pump down of ~6 s makes it possible to achieve a base pressure of <10^{-8} Torr. Before UVPD measurements, three or five consecutive hydration states are isolated for ions with n < 200 or $n \geq 200$, respectively, by applying a stored waveform inverse Fourier transform excitation. All mass spectrometric data are acquired with the MIDAS acquisition system.

In addition to UV initiated events, blackbody infrared radiative dissociation (BIRD) occurs as the ions are stored in the Penning trap for 0.1 – 2.0 s (see **Figure S1**). Absorption of blackbody photons emitted from the surrounding cell walls and copper jacket increases the internal energy of the stored ions and causes sequential water molecule loss to occur. For UVPD measurements, the precursor ion distribution is irradiated for 0.1 – 2.0 s with laser light from an EX50 Excimer Laser (**Figure S12**; GAM Laser Inc., Orlando, FL). However, there is no influence of the irradiation time on the number of water molecules lost upon absorption of one UV photon (also see **Irradiation Time Dependence**), consistent with previous findings.[5] The firing sequence of the laser is synchronized with the FT-ICR operation via a function generator, which is triggered by a voltage switch during the predefined ion storage time. The irradiation time is chosen in order to maximize product ion intensity and minimize the broadening of the ion distribution due to BIRD. The laser is operated at 250 Hz with ~5 W power for 193.3 ± 0.5 nm (6.41 ± 0.02 eV) and ~10 W for 248 ± 0.2 nm (4.991 ± 0.004 eV). Whereas 193 nm laser light was used for all ions, 248 nm photons were used to excite hydrated $(Phe+H)^+$, anilinium, Fe^{2+} and $[Ru(NH_3)_6]^{3+}$ ions that absorb at this wavelength. The laser

light is directed over two aluminum-coated mirrors, focused through a CaF_2 lens, and aligned through a CaF_2 window into the mass spectrometer (see **Figure S12**; for simplicity only one mirror is shown). Precursor and product ions are detected 0.5 s, 1.0 s and 1.5 s after irradiance with UV laser light for $n < 100$, $100 \leq n \leq 300$ and $n > 300$, respectively (also see section **Kinetic Shift**). Statistical uncertainties for the number of water molecules that are lost from the cluster are smaller than ±0.1 for $n < 150$ and are approximately ±0.3 for $n = 400$. The reported sequential water binding enthalpies are averaged between clusters with $\langle n \rangle$ and $\langle n \rangle - \langle x \rangle$ water molecules. In **Figure 6**, $\Delta H_{n,n-1}$ are shown as a function of $\langle n \rangle - \langle x \rangle/2$, the average value of the two known cluster sizes, and the cluster size uncertainty of $\pm\langle x \rangle/2$ is included in **Figure 6**.

Computational Modeling. Thermodynamic modeling for the relaxation process of clusters is required to infer binding enthalpies from UVPD measurements. All temperatures used for the modeling of the water evaporation process are effective temperatures and are parameters used to describe the average internal energy distribution of clusters and water molecules. Because the precursor ions are thermalized to approximately 133 K in the ICR cell, their internal energy prior to photon absorption is $U(133\ K)$. If there is full internal conversion, the absorbed photon energy will result in a change of the internal energy of the cluster described by **Equation 1**:

$$U_0(T_0^*) = U(133\ K) + h\nu \quad (1)$$

where the energy and effective temperature after photoexcitation are given by $[(U_0(T_0^*)]$ and T_0^*, respectively, and the photon energy is $h\nu$. The loss of water molecules from a cluster containing n water molecules will remove energy from the system thereby decreasing its effective temperature. Additionally, we assume that the energy gained by

the product cluster after water evaporation due to recoil of the ejected water molecule is negligible. The excess energy goes into overcoming the water molecule binding energy ($E_{n,n-1}$), and also partitions into the translational, rotational and vibrational modes of ejected water molecules (E_{VRT}). For the evaporated water molecules, vibrational excitations are neglected because the population of excited vibrational modes is lower than 4%. Consequently, the relation in **Equation 2**:

$$U_i(T_i^*) = U_{i-1}(T_{i-1}^*) - E_{n,n-1} - E_{VRT,i-1} \qquad (2)$$

connects the internal energy of consecutive hydration states during the evaporation process. The index i can take values between 1 and x, where x is the total number of lost water molecules. An average binding energies ($\langle E_{n,n-1}\rangle$) between the precursor size $\langle n\rangle$ and product size $\langle n\rangle - \langle x\rangle$ is calculated. The translational/rotational energy release given by the Klots evaporation model is used, *i.e.*

$$\langle E_{VRT}\rangle = \frac{5}{2}k_bT^* \qquad (3)$$

($k_b$is the Boltzmann constant).[6] The energy release term $\frac{5}{2}k_bT^*$ in the Klots model is the sum of the average rotational and average translational energy of $\frac{3}{2}k_bT^*$ and k_bT^* ,respectively and was derived for non-linear molecules. **Equation 1**, **Equation 2** and **Equation 3** together with the energy conservation relation in **Equation 4**:

$$h\nu = x \cdot \langle E_{n,n-1}\rangle + \sum_{i=1}^{x} E_{RT,i} \qquad (4)$$

define a series of equations that can be iteratively solved to find a solution for all T_i^* and $\langle E_{n,n-1}\rangle$. The internal energy of a cluster is calculated as a function of effective temperature by employing the partition function of uncoupled harmonic oscillators and using unscaled vibrational frequencies from B3LYP/LACVP**++ computations of

$Ca^{2+}(H_2O)_{14}$. The number of degrees of freedom are scaled accordingly. The use of other $Ca^{2+}(H_2O)_{14}$ isomers, larger Ca^{2+} clusters, other hydrated ions or different levels of theory affect the results by at maximum 0.8 kJ/mol. In addition to this absolute uncertainty, the maximum width of the excitation laser and the Klots evaporation model have uncertainties of ±2.1 (for 193 nm) and ±1.2 kJ/mol (**Figure S6**), respectively. The latter value is determined from the maximum difference in $\Delta H_{n,n-1}$ values obtained from measurements of the hydration enthalpy of the same ion with the same number of water molecules at both 248 and 193 nm. This results in an estimated absolute uncertainty for $\Delta H_{n,n-1}$ of less than 4.2 kJ/mol, which is comparable to or better than the uncertainties of other methods for determining the sequential water molecule binding energies of much smaller water clusters.[4,7–12]

The set of equations is solved iteratively in a newly developed Matlab R2015b program. A first guess of $\langle E_{n,n-1}\rangle$ is used to start the iterative procedure. The resulting effective temperatures and $\langle E_{n,n-1}\rangle$ are compared to $h\nu$ using **Equation 4**. In the next step, $\langle E_{n,n-1}\rangle$ is adjusted and this process is repeated until **Equation 4** is fulfilled. The calculation of $\langle E_{n,n-1}\rangle$ is repeated for all possible fragmentation channels from the precursor to the product distribution and the results are weighted by the measured ion abundances. The $\langle E_{n,n-1}\rangle$ values are converted using **Equation 5**:

$$\Delta H_{n,n-1} = \langle E_{n,n-1}\rangle + k_b T \qquad (5)$$

into binding enthalpies ($\Delta H_{n,n-1}$). **Equation 5** is derived under the assumption that the vibrational energy lost upon water loss from the precursor is equal to the gained translational, rotational, and vibrational energy of the products.[5]

For the TLDM model, the relation in **Equation 6** is used:[13]

$$\Delta G_{n,n-1} = \frac{z^2e^2}{8\pi\varepsilon_0}(1-\varepsilon^{-1})\left(\frac{4\pi\rho N_a}{3M}\right)^{\frac{1}{3}}\left[(n+a_i-1)^{-\frac{1}{3}}-(n+a_i)^{-\frac{1}{3}}\right]+$$
$$4\pi\gamma\left(\frac{3M}{4\pi\rho N_a}\right)^{2/3}\left[(n+a_i-1)^{\frac{2}{3}}-(n+a_i)^{\frac{2}{3}}\right]-k_bT\cdot ln\left(\frac{p}{p^0}\right) \quad (6)$$

where, z, ε, γ, ρ, $ln(p/p^0)$ and M are the charge state of the cluster, relative permittivity, surface energy, density, logarithm of the partial pressure relative to the standard pressure and molecular weight of water, respectively. The elementary charge, Avogadro constant, vacuum permittivity and a parameter to take the size of the ion into account are abbreviated by e, N_a, ε_0 and a_i, respectively. For all calculations, $a_i = 0$ is used. The thermodynamic relations $S = -\partial G/\partial T$ and $H = G + TS$ will give $\Delta H_{n,n-1}$ from **Equation 6** as a function of cluster size n. TLDM parameters for 133 K are given in **Table 1**.

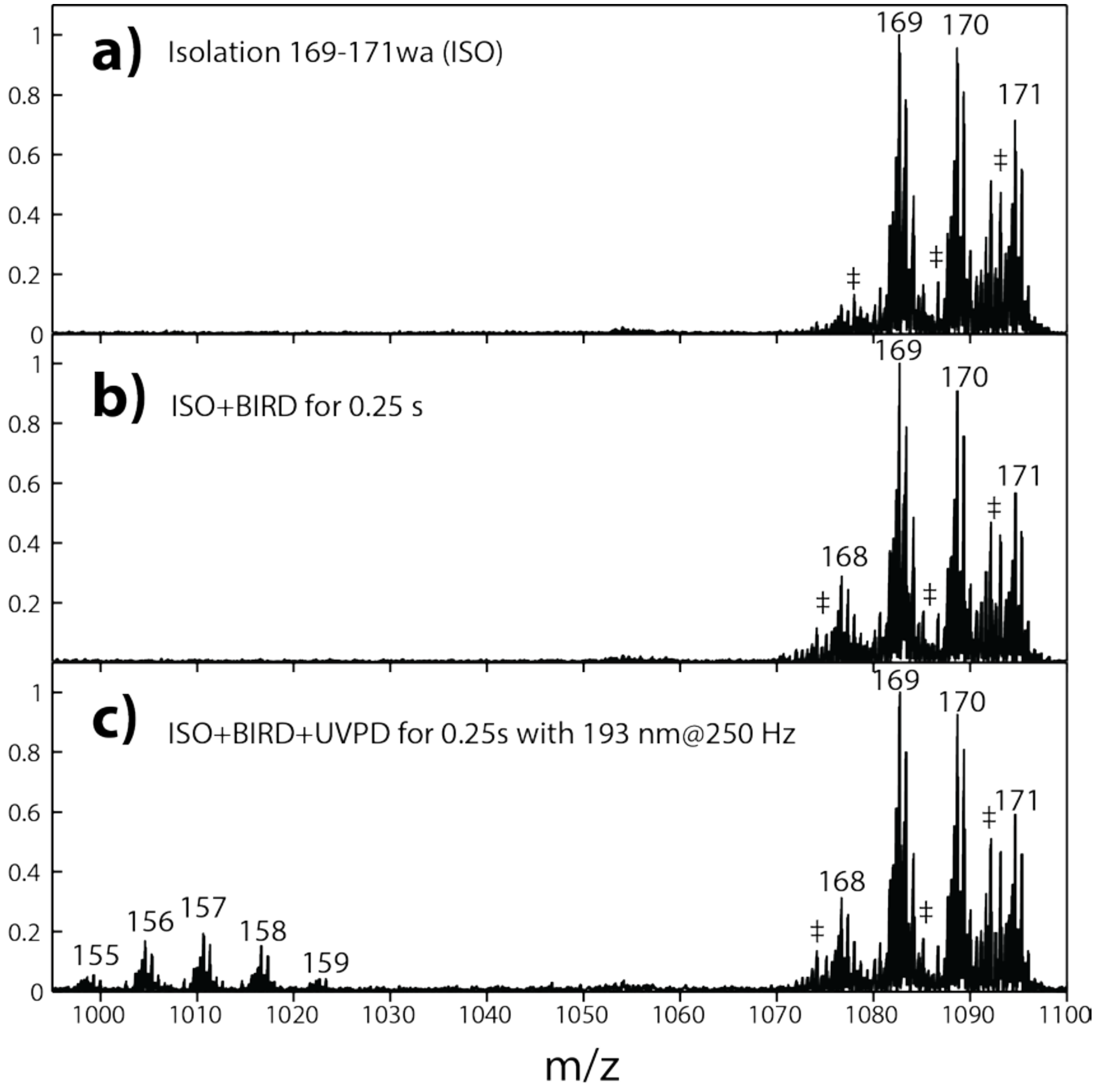

Figure S1. Typical experimental sequence: a) Isolation and subsequent detection of $[Ru(NH_3)]_6^{3+}{\cdot}(H_2O)_{169\text{-}171}$ in a nitrogen-cooled Penning trap. b) Storing the ion ensemble for 0.25 s in the Penning trap yields the shown mass spectrum with the BIRD product $[Ru(NH_3)]_6^{3+}{\cdot}(H_2O)_{168}$. c) UVPD mass spectrum with 193 nm@250Hz for 0.25 s yields the ions $[Ru(NH_3)]_6^{3+}{\cdot}(H_2O)_{155\text{-}159}$ and $[Ru(NH_3)]_6^{3+}{\cdot}(H_2O)_{168}$ is formed due to BIRD. (‡) $[Ru(NH_3)]_6^{2+}{\cdot}(H_2O)_n$.

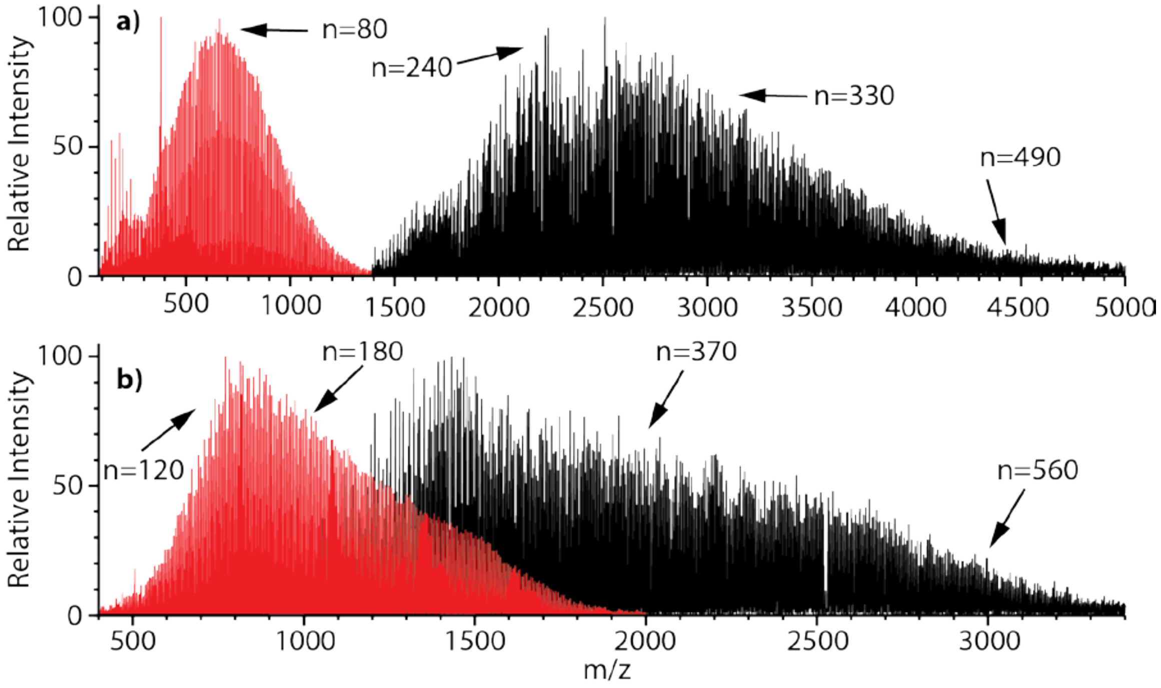

Figure S2. Overlay of two typical nanoESI mass spectra of (a) $Cu^{2+}\cdot(H_2O)_n$ and (b) $[Ru(NH_3)_6]^{3+}\cdot(H_2O)_n$ optimized for small (red) and large (black) clusters. Some cluster sizes *n* are labeled and highlighted with arrows.

Isotope Distribution Calculations. In order to unambiguously assign the peaks in the mass spectra, isotope distribution simulations for a couple of peaks in precursor and product distribution are performed.[14] For this purpose an isotope simulation code is developed in Matlab R2013b. The code uses the masses and isotope abundances from ref.[15] . Only exact masses are used, taking mass defects into account. The final mass is corrected for the addition or loss of electrons. The probability P is calculated from the expression $P = \prod_i P(E_i)^{\nu_i}$, where $P(E_i)$ is the probability for a given isotopic configuration of element E_i, ν_i is the number of atoms of E_i and the index i runs over all

elements in the compound. $P(E_i)$ are multinomial distributions of the isotopes of the element E_i.

The logarithmic forms of these probability expressions are used to avoid a numerical explosion. The isotopes are generated by combined rationale and random excitation of nucleons. All single and double nucleon excitations to the second most abundant isotope (if existing) are performed. Additionally, a series of random nucleon excitations for an excitation level of up to 10 can be performed generating up to $2 \cdot 10^6$ random isotopic configurations. For the systems discussed here 5 excitations are sufficient. The exact masses and probabilities for all generated isotopomers are calculated and visualized as a stick spectrum. The stick spectrum can be convolved with Lorentzian or Gaussian functions. Isotope distributions for Angiotensin II, Ubiquitin, $Al^{3+} \cdot (H_2O)_{200}$ and $Cu^{2+} \cdot (H_2O)_{200}$ agreed with results from the ChemCalc online tool.[16]

Typical results for clusters of the precursor (and product) distribution in UVPD experiments are shown in **Figure S3**, **Figure S4** and **Figure S5** for $(Phe+H)^+ \cdot (H_2O)_{187/200}$ $Cu^{2+} \cdot (H_2O)_{200}$ and $[Ru(NH_3)]_6^{3+} \cdot (H_2O)_{186/200}$, respectively. The mass accuracy of the experimental peaks compared to theoretical values and the used Gaussian width is given, too. For the experimental spectra a Hanning apodization is applied and two zero fills are added to the $(1\text{-}2)10^6$ recorded data points. In **Figure S3c** a not identified chemical noise peak is observable. This noise peak is easily discriminated from the $(Phe+H)^+ \cdot (H_2O)_{200}$ isotope distribution and is absent in the UVPD products (**Figure S3a**).

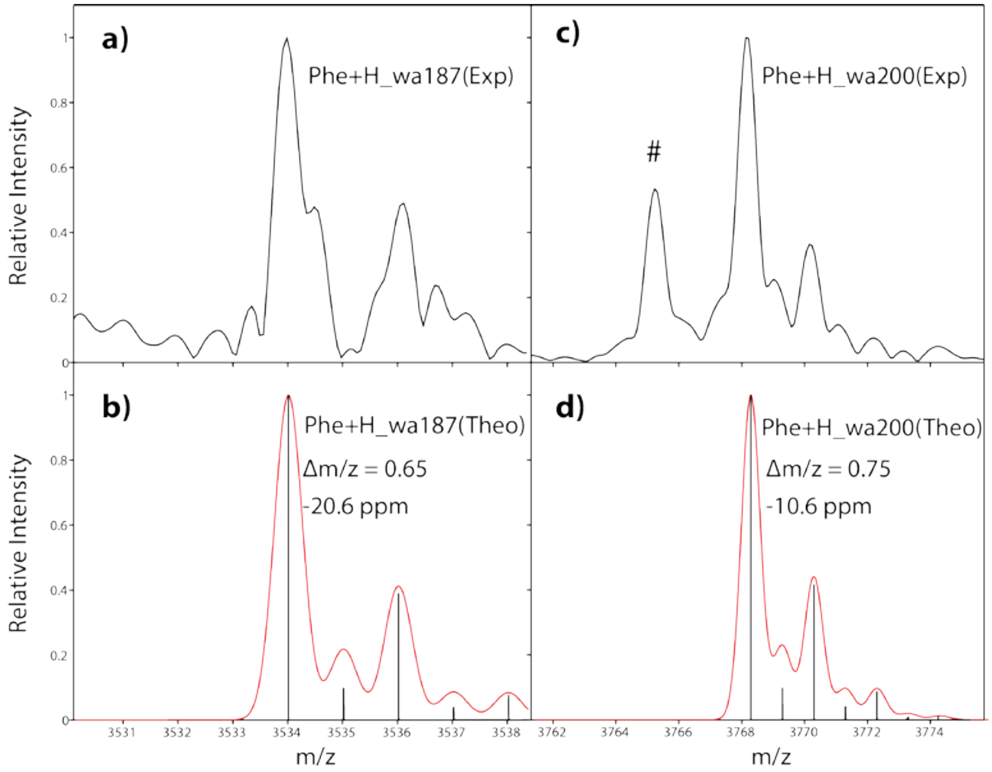

Figure S3. Comparison of the experimental (a+c) and theoretical (b+d) isotope distribution of $(Phe+H)^{+}\cdot(H_2O)_{187}$ (a+c) and $(Phe+H)^{+}\cdot(H_2O)_{200}$ (b+d). The full width at half maximum (FWHM) of the Gaussian functions is given as Δm/z values. The mass error is included in (b) and (d). (#) Unidentified chemical noise.

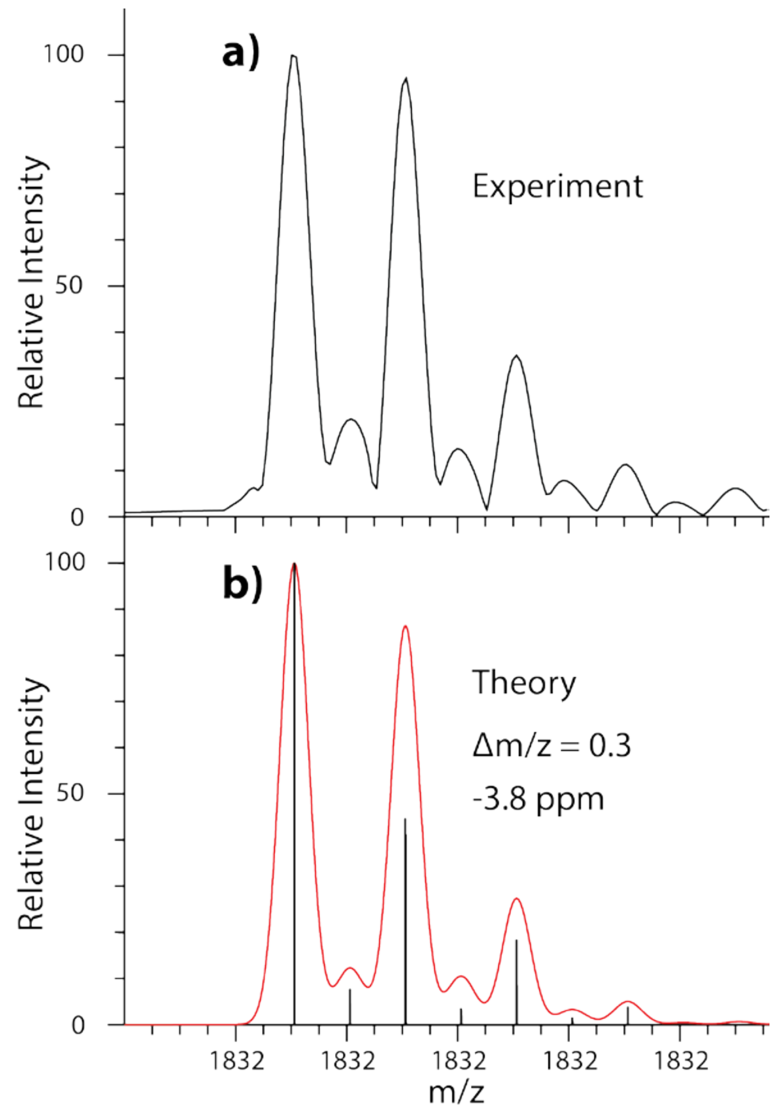

Figure S4. Comparison of the experimental (a) and theoretical (b) isotope distribution of $Cu^{2+}\cdot(H_2O)_{200}$. The FWHM of the Gaussian functions is given as Δm/z value. The mass error is included in (b).

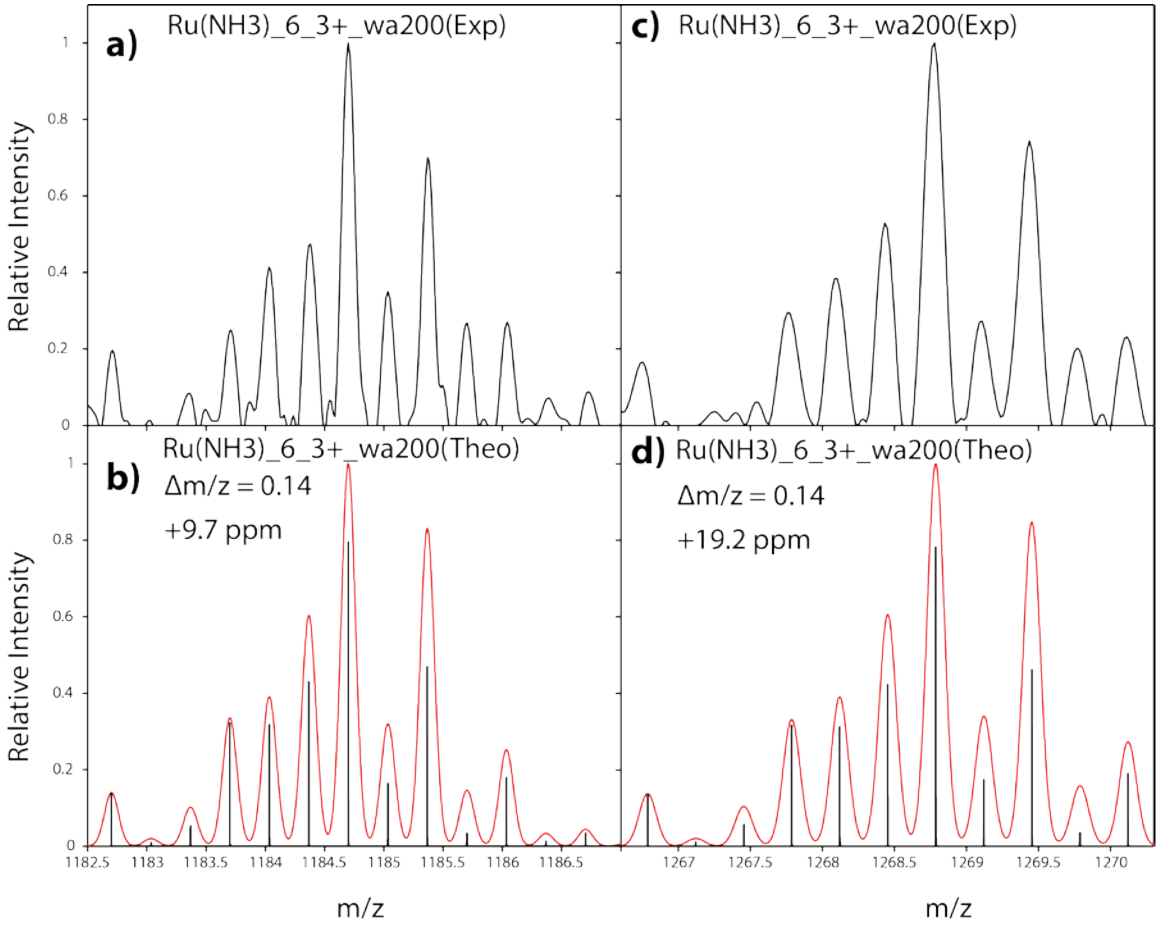

Figure S5. Comparison of the experimental (a+c) and theoretical (b+d) isotope distribution of $[Ru(NH_3)_6]^{3+}\cdot(H_2O)_{187}$ (a+c) and $[Ru(NH_3)_6]^{3+}\cdot(H_2O)_{200}$ (b+d). The full width at half maximum (FWHM) of the Gaussian functions is given as Δm/z values. The mass error is included in (b) and (d).

Irradiation Time Dependence. Results by Williams and coworkers indicate that UVPD results of hydrated ions are to a good approximation independent of the laser irradiation time and only influence the photoproduct yield.[5] In order to test if the laser irradiation time does not influence the results, we performed UVPD experiments for $(Phe+H)^+\cdot(H_2O)_{45}$ with varying laser irradiation time. For $(Phe+H)^+\cdot(H_2O)_{45}$, the number of lost water molecules are 13.8, 13.7, 13.8 and 13.8 after 100, 250, 500 and 1000 ms of irradiation with 193 nm laser light. This is consistent with previous results.[5,17] This

indicates that the difference in BIRD rates for precursor and product ion distributions are negligibly small and that laser irradiation time does not significantly influence ⟨*x*⟩.

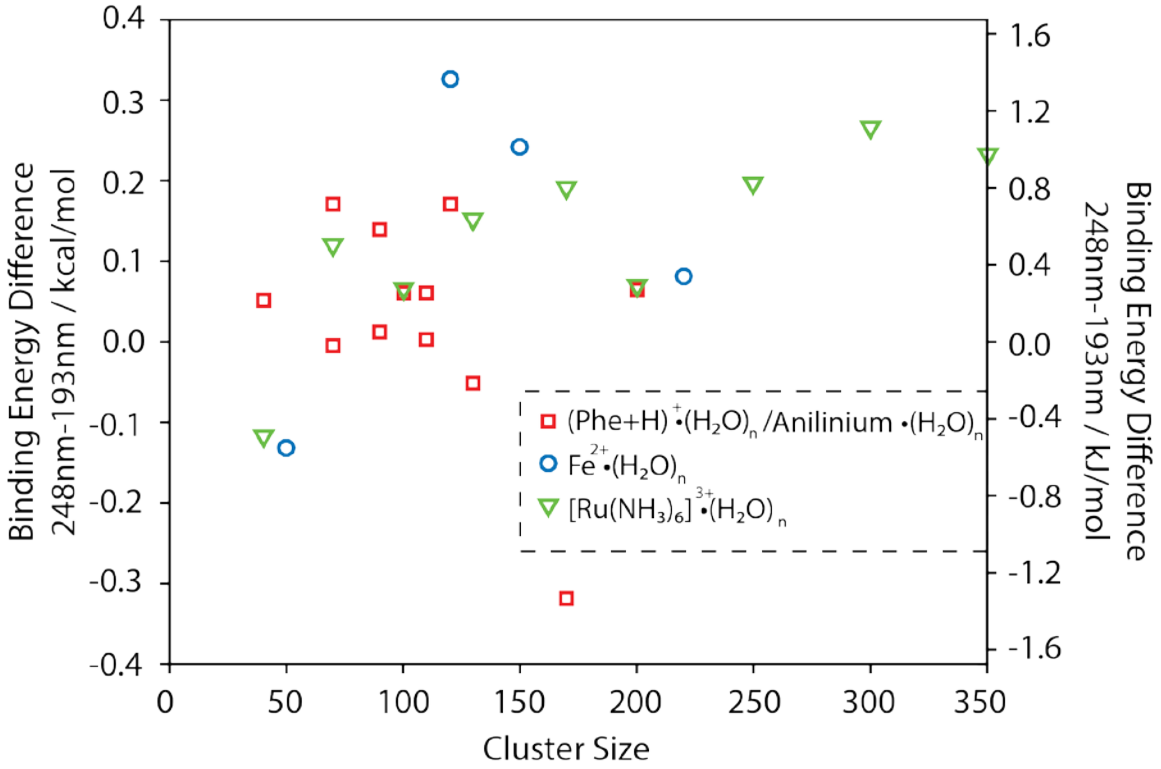

Figure S6. Difference between the binding energies determined from 248 nm and 193 nm UVPD experiments as a function of median cluster size. For singly charged clusters $(Phe{+}H)^{+}{\cdot}(H_2O)_n$ and anilinium$\cdot(H_2O)_n$ were used.

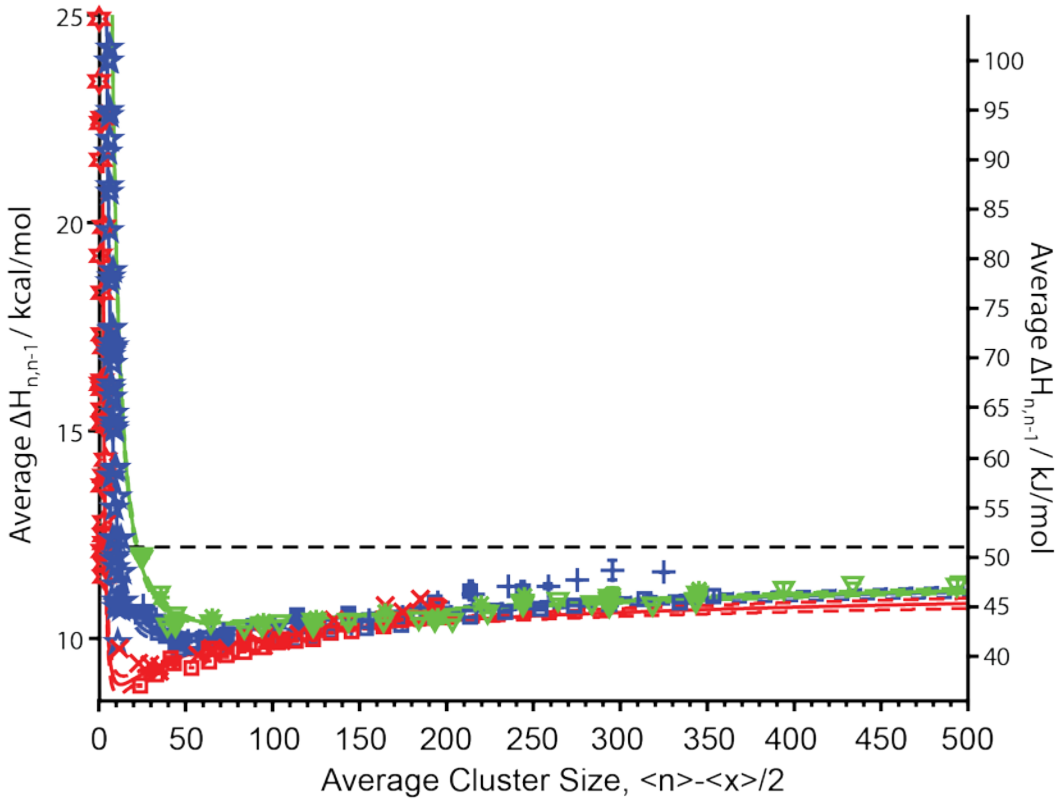

Figure S7. The average sequential binding enthalpy $\Delta H_{n,n-1}$ in kcal/mol (left axis) and kJ/mol (right axis) deduced from UVPD measurements for hydrated PTMA, $(Phe+H)^{+}$, Anilinium (□);Cu^{2+}, Co^{2+} (□); Fe^{2+}, Mn^{2+} (■); $[Co(NH_3)_6]^{3+}$, $[Cr(NH_3)_6]^{3+}$, $[Ru(NH_3)_6]^{3+}$ (▽) ions upon 193 nm photon absorption and $(Phe+H)^{+}$, Anilinium (✕);Fe^{2+} (+); $[Ru(NH_3)_6]^{3+}$ (✳) upon 248 nm photon absorption as a function of as a function of $\langle n \rangle - \langle x \rangle/2$. The sublimation enthalpy $\Delta H_{sub} = 51.0$ kJ/mol of bulk water ice at 133 K and the vaporization enthalpy ΔH_{vap} = 43.1 kJ/mol of bulk water at 313 K are depicted as dashed black horizontal line. The TLDM at 133 K and the fitted TLDM are shown as dashed and solid red, blue and green lines for mono-, di-, and trivalent ions, respectively. Literature binding enthalpies for cluster with $\langle n \rangle - \langle x \rangle/2 \leq 12$ monovalent (✡) and divalent (★) ions are included in the figure.

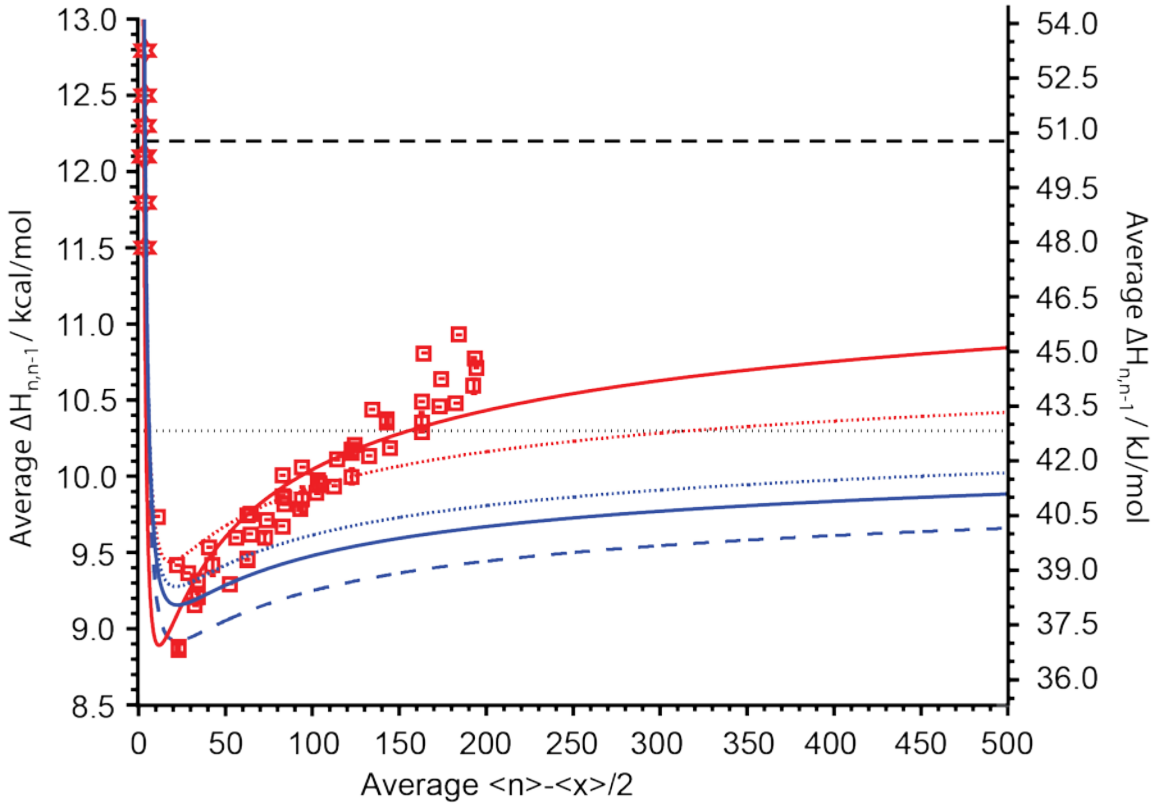

Figure S8. The average sequential binding enthalpy $\Delta H_{n,n-1}$ in kcal/mol (left axis) and kJ/mol (right axis) deduced from UVPD measurements for hydrated PTMA, $(Phe+H)^+$, Anilinium and $(Phe+H)^+$ (□) ions upon 193 nm and 248 nm photon absorption as a function of as a function of $\langle n \rangle - \langle x \rangle /2$. The sublimation enthalpy $\Delta H_{sub} = 51.0$ kJ/mol of bulk water ice at 133 K and the vaporization enthalpy ΔH_{vap} = 43.1 kJ/mol of bulk water at 313 K are depicted as dashed and dotted black horizontal lines, respectively. The TLDM at 133 K and the fitted TLDM are shown as dotted and solid redlines, respectively. TLDM predictions for 313 K, 298 K and 273 K parameters for liquid water are shown as dashed, solid and dotted blue lines, respectively. The mean difference of the 313 K, 298 K and 273 K TLDM to the experimental data points is 4.69 kJ/mol, 2.47 kJ/mol and 2.01 kJ/mol, respectively. The mean difference of the 133 K TLDM to the experimental data points is 1.25 kJ/mol. Literature binding enthalpies for cluster with $\langle n \rangle - \langle x \rangle /2 \leq 12$ monovalent (✡) ions are included in the figure.

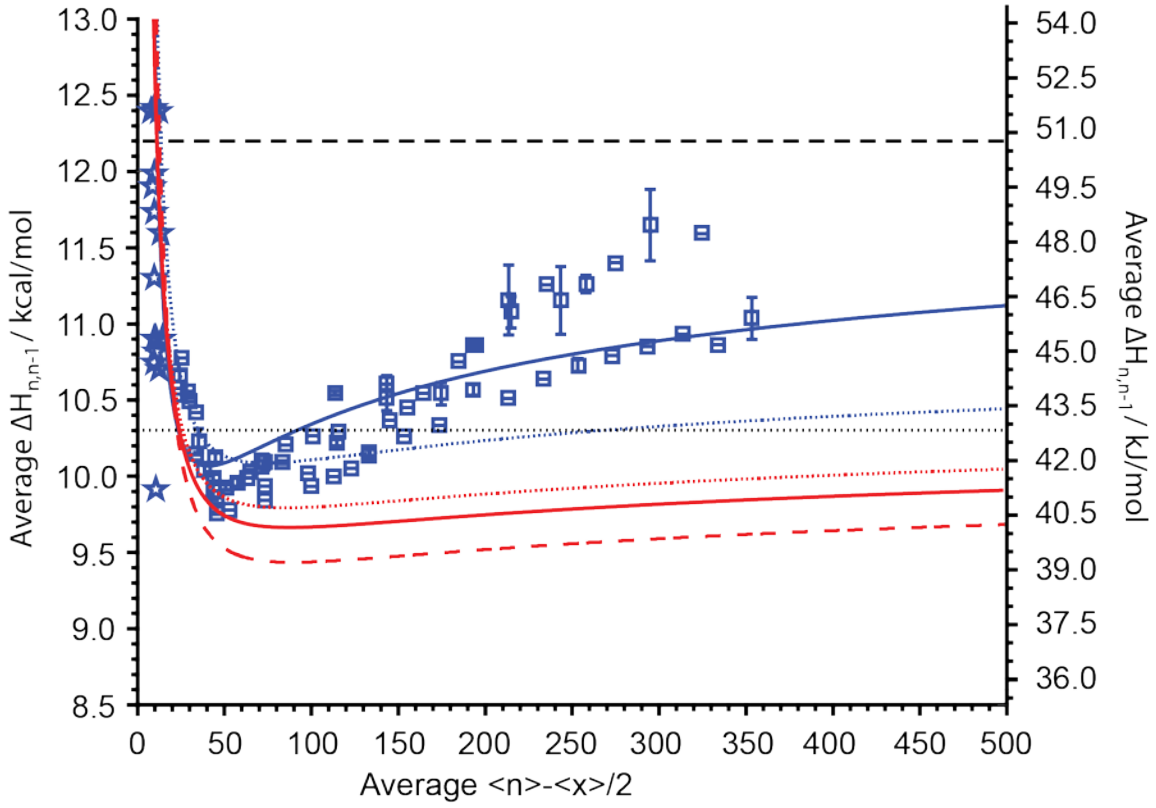

Figure S9. The average sequential binding enthalpy $\Delta H_{n,n-1}$ in kcal/mol (left axis) and kJ/mol (right axis) deduced from UVPD measurements for hydrated Cu^{2+}, Co^{2+}, Fe^{2+} and Mn^{2+} (□) ions upon 193 nm and 248 nm photon absorption as a function of as a function of $\langle n \rangle - \langle x \rangle/2$. The sublimation enthalpy $\Delta H_{sub} = 51.0$ kJ/mol of bulk water ice at 133 K and the vaporization enthalpy ΔH_{vap} = 43.1 kJ/mol of bulk water at 313 K are depicted as dashed and dotted black horizontal lines, respectively. The TLDM at 133 K and the fitted TLDM are shown as dotted and solid blue lines, respectively. TLDM predictions for 313 K, 298 K and 273 K parameters for liquid water are shown as dashed, solid and dotted red lines, respectively. The mean difference of the 313 K, 298 K and 273 K TLDM to the experimental data points is 5.52 kJ/mol, 3.34 kJ/mol and 2.89 kJ/mol, respectively. The mean difference of the 133 K TLDM to the experimental data points is 1.92 kJ/mol. Literature binding enthalpies for cluster with $\langle n \rangle - \langle x \rangle/2 \leq 12$ divalent (☆) ions are included in the figure.

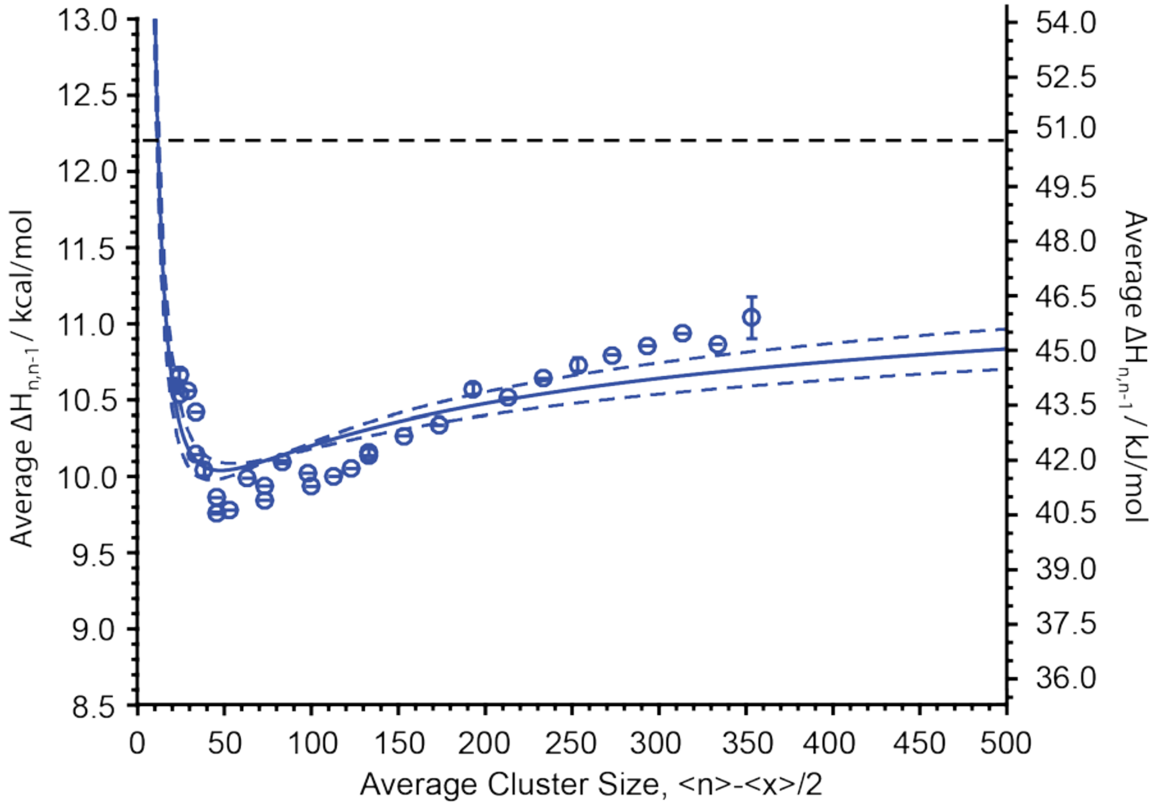

Figure S10. The average sequential binding enthalpy $\Delta H_{n,n-1}$ in kcal/mol (left axis) and kJ/mol (right axis) deduced from UVPD measurements for hydrated Cu^{2+} and Co^{2+} (○) ions upon 193 nm photon absorption as a function of as a function of $\langle n\rangle - \langle x\rangle/2$. The sublimation enthalpy $\Delta H_{sub} = 51.0$ kJ/mol of bulk water ice at 133 K are shown as dashed black horizontal line. The fitted TLDM and the corresponding uncertainties are shown as solid and dashed blue lines, respectively.

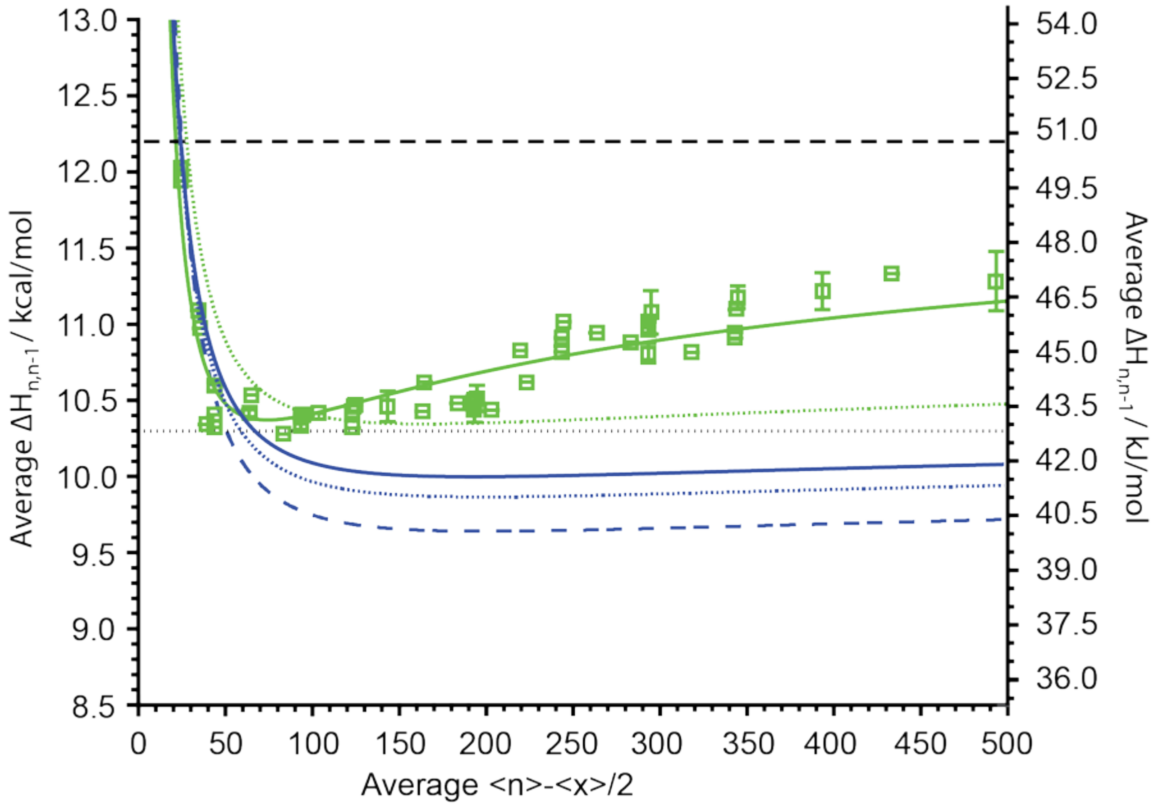

Figure S11. The average sequential binding enthalpy $\Delta H_{n,n-1}$ in kcal/mol (left axis) and kJ/mol (right axis) deduced from UVPD measurements for hydrated $[Co(NH_3)_6]^{3+}$, $[Cr(NH_3)_6]^{3+}$, and $[Ru(NH_3)_6]^{3+}$ (□) ions upon 193 nm and 248 nm photon absorption as a function of as a function of $\langle n \rangle - \langle x \rangle/2$. The sublimation enthalpy $\Delta H_{sub} = 51.0$ kJ/mol of bulk water ice at 133 K and the vaporization enthalpy ΔH_{vap} = 43.1 kJ/mol of bulk water at 313 K are depicted as dashed and dotted black horizontal lines, respectively. The TLDM at 133 K and the fitted TLDM are shown as dotted and solid green lines, respectively. TLDM predictions for 313 K, 298 K and 273 K parameters for liquid water are shown as dashed, dotted and solid blue lines, respectively. The mean difference of the 313 K, 298 K and 273 K TLDM to the experimental data points is 5.40 kJ/mol, 3.34 kJ/mol and 2.92 kJ/mol, respectively. The mean difference of the 133 K TLDM to the experimental data points is 2.05 kJ/mol.

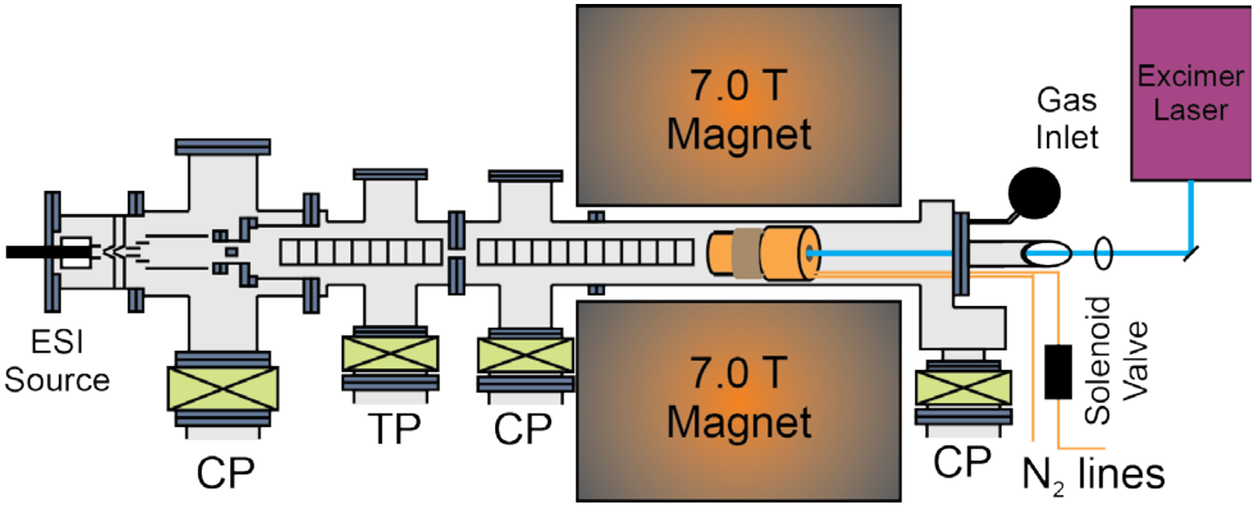

Figure S12. Schematic diagram of the home-built 7 T FT-ICR mass spectrometer at Berkeley configured for UVPD experiments. CP and TP refer to cryogenic and turbo molecular pumps, respectively.

Table S1. The calculated average sequential binding enthalpies, $\Delta H_{n,n-1}$, in kcal/mol and kJ/mol at 133 K and the average number of water molecules lost, ⟨*x*⟩, extracted from the experimental UVPD measurements as a function of ion identity, charge state, median cluster size and laser wavelength. All uncertainties reported are standard deviations determined from triplicate measurements.

**Ion**	**Median cluster size**	**$\Delta H_{n,n-1}$ / kcal/mol**	**$\Delta H_{n,n-1}$ / kJ/mol**	**⟨*x* ⟩**	**Wavelength**
PheAla+	30	8.86±0.01	37.09±0.04	13.83±0.001	193 nm
PheAla+	40	9.15	38.30	13.84	193 nm
PheAla+	50	9.42±0.07	39.43±0.30	13.75±0.10	193 nm
PheAla+	60	9.30	38.90	14.08	193 nm
PheAla+	70	9.45	39.55	14.01	193 nm
PheAla+	80	9.59±0.03	40.14±0.14	13.92±0.04	193 nm
PheAla+	90	9.68	40.49	13.91	193 nm
PheAla+	100	9.79±0.01	40.94±0.03	13.86±0.01	193 nm
PheAla+	110	9.89	41.37	13.79	193 nm

PheAla+	120	9.94	41.59	13.79	193 nm
PheAla+	130	10.00±0.05	41.85±0.21	13.76±0.06	193 nm
PheAla+	140	10.14	42.41	13.63	193 nm
PheAla+	152	10.19	43.65	13.58	193 nm
PheAla+	170	10.36±0.06	43.34±0.25	13.62±0.08	193 nm
PheAla+	180	10.46	43.75	13.60	193 nm
PheAla+	190	10.48	43.86	13.54	193 nm
PheAla+	200	10.59±0.05	44.32±0.20	13.49±0.06	193 nm
Aniline+	70	9.75	40.80	13.66	193 nm
Aniline+	90	9.87	41.32	13.68	193 nm
Aniline+	110	9.96	41.66	13.71	193 nm
Aniline+	130	10.15	42.48	13.57	193 nm
Aniline+	150	10.38	43.41	13.38	193 nm
Aniline+	170	10.49	43.91	13.34	193 nm
Aniline+	200	10.78	45.11	13.25	193 nm
PTMA+	30	8.89	37.18	13.80	193 nm
PTMA+	48	9.54	39.90	13.60	193 nm
PTMA+	70	9.46	39.57	14.00	193 nm
PTMA+	90	10.01	41.88	13.54	193 nm
PTMA+	110	9.97	41.73	13.71	193 nm
PTMA+	130	10.18	42.58	13.56	193 nm
PTMA+	150	10.36	43.33	13.41	193 nm
PTMA+	170	10.29	43.06	13.49	193 nm
PheAla+	40	9.21±0.03	38.52±0.12	11.02±0.03	248 nm
PheAla+	70	9.62	40.27	10.94	248 nm
PheAla+	90	9.82	41.07	10.86	248 nm
PheAla+	100	9.85±0.08	41.21±0.35	10.89±0.10	248 nm
PheAla+	110	9.95	41.63	10.81	248 nm
PheAla+	120	10.11	42.30	10.74	248 nm
Aniline+	16	9.74	40.75	9.59	248 nm
Aniline+	28	9.42	39.41	10.42	248 nm
Aniline+	34	9.37	39.19	10.66	248 nm
Aniline+	40	9.31	38.96	10.87	248 nm
Aniline+	62	9.60	40.16	10.88	248 nm
Aniline+	70	9.76	40.82	10.77	248 nm
Aniline+	80	9.72	40.66	10.93	248 nm
Aniline+	90	9.86	41.26	10.89	248 nm
Aniline+	100	10.06	42.10	10.74	248 nm
Aniline+	110	9.95	41.64	10.84	248 nm
Aniline+	120	10.11	42.31	10.72	248 nm
Aniline+	130	10.21	42.70	10.66	248 nm
Aniline+	140	10.44	43.68	10.56	248 nm
Aniline+	170	10.81	45.24	10.52	248 nm
Aniline+	180	10.64	44.53	10.65	248 nm
Aniline+	190	10.93	45.74	10.52	248 nm

Aniline+	200	10.72	44.83	10.58	248 nm
Cu2+	30	10.66±0.03	44.59±0.15	11.57±0.15	193 nm
Cu2+	35	10.56	44.17	11.91	193 nm
Cu2+	40	10.42	43.58	12.20	193 nm
Cu2+	45	10.05±0.04	42.03±0.18	12.72±0.02	193 nm
Cu2+	52	9.75±0.01	40.83±0.03	13.21±0.02	193 nm
Cu2+	60	9.78	40.91	13.37	193 nm
Cu2+	70	9.99	41.79	13.28	193 nm
Cu2+	80	9.94	41.57	13.46	193 nm
Cu2+	90	10.09±0.01	42.22±0.04	13.37±0.01	193 nm
Cu2+	107	9.93	41.56	13.67	193 nm
Cu2+	120	10.00	41.83	13.66	193 nm
Cu2+	130	10.05	42.05	13.65	193 nm
Cu2+	140	10.16±0.03	42.51±0.11	13.57±0.05	193 nm
Cu2+	160	10.27	42.95	13.50	193 nm
Cu2+	180	10.33	43.24	13.49	193 nm
Cu2+	200	10.57±0.04	44.21±0.15	13.46±0.04	193 nm
Cu2+	220	10.51	43.97	13.38	193 nm
Cu2+	240	10.64	44.51	13.47	193 nm
Cu2+	260	10.73±0.04	44.89±0.18	13.44±0.04	193 nm
Cu2+	280	10.79	45.15	13.37	193 nm
Cu2+	300	10.85	45.40	13.30	193 nm
Cu2+	320	10.93	45.73	13.24	193 nm
Cu2+	340	10.86	45.45	13.32	193 nm
Cu2+	360	11.04±0.14	46.17±0.58	13.25±0.10	193 nm
Co2+	30	10.54	44.10	11.72	193 nm
Co2+	40	10.14	42.43	12.51	193 nm
Co2+	52	9.86	41.24	13.15	193 nm
Co2+	80	9.84	41.18	13.56	193 nm
Co2+	105	10.02	41.92	13.54	193 nm
Co2+	140	10.13	42.40	13.58	193 nm
Co2+	180	10.33	43.23	13.49	193 nm
Mn2+	50	9.90±0.06	41.43±0.24	13.05±0.06	193 nm
Mn2+	78	10.06	42.09	13.35	193 nm
Mn2+	122	10.29	43.07	13.39	193 nm
Mn2+	150	10.52±0.09	44.01±0.36	13.25±0.08	193 nm
Mn2+	200	10.86±0.02	45.43±0.08	13.27±0.04	193 nm
Mn2+	250	11.15±0.22	46.66±0.93	13.12±0.17	193 nm
Fe2+	50	9.99±0.02	41.80±0.09	12.95±0.02	193 nm
Fe2+	80	10.09±0.05	42.22±0.19	13.31±0.05	193 nm
Fe2+	120	10.55±0.01	44.13±0.06	13.13±0.01	193 nm
Fe2+	150	10.61±0.05	44.39±0.22	13.17±0.03	193 nm
Fe2+	220	11.16±0.23	46.68±0.96	12.94±0.19	193 nm
Fe2+	30	10.78	45.08	9.31	248 nm
Fe2+	35	10.49	43.88	9.63	248 nm

Fe2+	40	10.23±0.08	42.82±0.33	9.91±0.06	248 nm
Fe2+	50	10.12±0.03	42.35±0.13	10.18±0.03	248 nm
Fe2+	56	9.93±0.02	41.54±0.07	10.44±0.02	248 nm
Fe2+	63	9.96±0.03	41.67±0.11	10.49±0.02	248 nm
Fe2+	70	10.03±0.01	41.97±0.04	10.50±0.01	248 nm
Fe2+	77	10.11	42.28	10.49	248 nm
Fe2+	90	10.21	42.70	10.55	248 nm
Fe2+	106	10.26	42.95	10.55	248 nm
Fe2+	120	10.22±0.02	42.76±0.09	10.61±0.02	248 nm
Fe2+	150	10.37	43.38	10.56	248 nm
Fe2+	160	10.45	43.72	10.52	248 nm
Fe2+	170	10.54	44.17	10.52	248 nm
Fe2+	180	10.54±0.07	44.10±0.31	10.51±0.05	248 nm
Fe2+	190	10.75	44.98	10.45	248 nm
Fe2+	200	10.86	45.45	10.48	248 nm
Fe2+	220	11.08±0.10	46.34±0.43	10.33±0.07	248 nm
Fe2+	240	11.26	47.09	10.32	248 nm
Fe2+	263	11.26±0.06	47.12±0.24	10.31±0.06	248 nm
Fe2+	280	11.40	47.70	10.20	248 nm
Fe2+	300	11.65±0.23	48.74±0.98	10.05±0.11	248 nm
Fe2+	330	11.59	48.50	10.07	248 nm
$[Ru(NH_3)_6]^{3+}$	30	12.03±0.04	50.32±0.16	10.75±0.03	193 nm
$[Ru(NH_3)_6]^{3+}$	40	11.09	46.41	11.76	193 nm
$[Ru(NH_3)_6]^{3+}$	45	10.34	43.27	12.59	193 nm
$[Ru(NH_3)_6]^{3+}$	50	10.32	43.20	12.70	193 nm
$[Ru(NH_3)_6]^{3+}$	70	10.42±0.02	43.58±0.10	12.88±0.02	193 nm
$[Ru(NH_3)_6]^{3+}$	90	10.28	43.01	13.21	193 nm
$[Ru(NH_3)_6]^{3+}$	100	10.34±0.03	43.26±0.12	13.21±0.03	193 nm
$[Ru(NH_3)_6]^{3+}$	110	10.42	43,58	13.16	193 nm
$[Ru(NH_3)_6]^{3+}$	130	10.32	43.18	13.35	193 nm
$[Ru(NH_3)_6]^{3+}$	150	10.46±0.10	43.77±0.42	13.30±0.10	193 nm
$[Ru(NH_3)_6]^{3+}$	170	10.43	43.64	13.36	193 nm
$[Ru(NH_3)_6]^{3+}$	190	10.48	43.86	13.36	193 nm
$[Ru(NH_3)_6]^{3+}$	200	10.44±0.08	43.70±0.36	13.43±0.08	193 nm
$[Ru(NH_3)_6]^{3+}$	210	10.44	43.67	13.44	193 nm
$[Ru(NH_3)_6]^{3+}$	230	10.62	44.45	13.42	193 nm
$[Ru(NH_3)_6]^{3+}$	250	10.82	45.27	13.27	193 nm
$[Ru(NH_3)_6]^{3+}$	270	10.95	45.80	13.23	193 nm
$[Ru(NH_3)_6]^{3+}$	290	10.88	45.54	13.17	193 nm
$[Ru(NH_3)_6]^{3+}$	300	10.81±0.06	45.24±0.27	13.32±0.06	193 nm
$[Ru(NH_3)_6]^{3+}$	325	10.82	45.26	13.33	193 nm
$[Ru(NH_3)_6]^{3+}$	350	10.95	46.80	13.21	193 nm
$[Co(NH_3)_6]^{3+}$	30	11.94	49.96	10.81	193 nm
$[Co(NH_3)_6]^{3+}$	50	10.59±0.03	44.32±0.11	12.45±0.03	193 nm
$[Co(NH_3)_6]^{3+}$	100	10.38±0.03	43.45±0.11	13.16±0.03	193 nm

$[Co(NH_3)_6]^{3+}$	130	10.47	43.79	13.20	193 nm
$[Co(NH_3)_6]^{3+}$	200	10.49±0.06	43.89±0.25	13.36±0.06	193 nm
$[Co(NH_3)_6]^{3+}$	250	10.91	45.65	13.15	193 nm
$[Co(NH_3)_6]^{3+}$	300	10.97±0.02	45.88±0.08	13.20±0.03	193 nm
$[Co(NH_3)_6]^{3+}$	350	11.10	46.43	13.07	193 nm
$[Co(NH_3)_6]^{3+}$	400	11.22±0.12	46.94±0.50	13.02±0.06	193 nm
$[Co(NH_3)_6]^{3+}$	440	11.33	47.42	12.87	193 nm
$[Co(NH_3)_6]^{3+}$	500	11.29±0.19	47.22±0.81	12.99±0.17	193 nm
$[Cr(NH_3)_6]^{3+}$	50	10.41	43.54	12.64	193 nm
$[Cr(NH_3)_6]^{3+}$	130	10.43	43.65	13.29	193 nm
$[Cr(NH_3)_6]^{3+}$	200	10.45	43.74	13.35	193 nm
$[Cr(NH_3)_6]^{3+}$	250	10.86	45.43	13.24	193 nm
$[Cr(NH_3)_6]^{3+}$	300	11.02	46.09	13.12	193 nm
$[Cr(NH_3)_6]^{3+}$	350	10.91	45.65	13.26	193 nm
$[Ru(NH_3)_6]^{3+}$	40	10.98	45.92	9.47	248 nm
$[Ru(NH_3)_6]^{3+}$	70	10.54	44.09	10.13	248 nm
$[Ru(NH_3)_6]^{3+}$	100	10.41±0.04	43.54±0.17	10.37±0.03	248 nm
$[Ru(NH_3)_6]^{3+}$	130	10.47	43.81	10.49	248 nm
$[Ru(NH_3)_6]^{3+}$	170	10.62	44.44	10.43	248 nm
$[Ru(NH_3)_6]^{3+}$	200	10.51±0.08	43.99±0.35	10.53±0.08	248 nm
$[Ru(NH_3)_6]^{3+}$	225	10.83	45.30	10.52	248 nm
$[Ru(NH_3)_6]^{3+}$	250	11.02	46.10	10.42	248 nm
$[Ru(NH_3)_6]^{3+}$	300	11.08±0.14	46.35±0.60	10.37±0.08	248 nm
$[Ru(NH_3)_6]^{3+}$	350	11.18±0.07	46.77±0.31	10.38±0.04	248 nm

References

1 W. A. Donald, R. D. Leib, M. Demireva, B. Negru, D. M. Neumark and E. R. Williams, *J. Am. Chem. Soc.*, 2010, **132**, 6904–6905.

2 M. F. Bush, R. J. Saykally and E. R. Williams, *Int. J. Mass Spectrom.*, 2006, **253**, 256–262.

3 D. R. Brown and R. R. Pavlis, *J. Chem. Educ.*, 1985, **62**, 807.

4 R. L. Wong, K. Paech and E. R. Williams, *Int. J. Mass Spectrom.*, 2004, **232**, 59–66.

5 W. A. Donald, R. D. Leib, M. Demireva, B. Negru, D. M. Neumark and E. R. Williams, *J. Phys. Chem. A*, 2011, **115**, 2–12.

6 C. E. Klots, *J. Chem. Phys.*, 1985, **83**, 5854.

7 S. E. Rodriguez-Cruz, R. A. Jockusch and E. R. Williams, *J. Am. Chem. Soc.*, 1998, **120**, 5842–5843.

8 S. E. Rodriguez-Cruz, R. A. Jockusch and E. R. Williams, *J. Am. Chem. Soc.*, 1999, **121**, 8898–8906.

9 T. E. Cooper and P. B. Armentrout, *Chem. Phys. Lett.*, 2010, **486**, 1–6.

10 M. Peschke, A. T. Blades and P. Kebarle, *J. Phys. Chem. A*, 1998, **102**, 9978–9985.

11 A. T. Blades and P. Kebarle, *J. Phys. Chem. A*, 2005, **109**, 8293–8298.

12 M. T. Rodgers and P. B. Armentrout, *J. Phys. Chem. A*, 1997, **101**, 1238–1249.

13 W. A. Donald and E. R. Williams, *J. Phys. Chem. A*, 2008, **112**, 3515–3522.

14 D. Valkenborg, I. Mertens, F. Lemière, E. Witters and T. Burzykowski, *Mass Spectrom. Rev.*, 2012, **31**, 96–109.

15 P. de Biévre and Taylor, P. D. P., *International Journal of Mass Spectrometry and Ion Processes*, 1993, **123**, 149–166.

16 L. Patiny and A. Borel, *J. Chem. Inf. Model.*, 2013, **53**, 1223–1228.

17 W. A. Donald, R. D. Leib, M. Demireva and E. R. Williams, *J. Am. Chem. Soc.*, 2011, **133**, 18940–18949.
